# Very long O‐antigen chains of *Salmonella* Paratyphi A inhibit inflammasome activation and pyroptotic cell death

**DOI:** 10.1111/cmi.13306

**Published:** 2021-01-17

**Authors:** Elli Mylona, Julia Sanchez‐Garrido, Trang Nguyen Hoang Thu, Sabina Dongol, Abhilasha Karkey, Stephen Baker, Avinash R. Shenoy, Gad Frankel

**Affiliations:** ^1^ Department of Life Sciences MRC Centre for Molecular Bacteriology and Infection, Imperial College London London UK; ^2^ Cambridge Institute of Therapeutic Immunology & Infectious Disease (CITIID), Department of Medicine University of Cambridge Cambridge UK; ^3^ Oxford University Clinical Research Unit The Hospital for Tropical Diseases Ho Chi Minh City Vietnam; ^4^ Oxford University Clinical Research Unit Patan Academy of Health Sciences Kathmandu Nepal; ^5^ Department of Infectious Disease MRC Centre for Molecular Bacteriology and Infection, Imperial College London London UK

**Keywords:** caspase‐4, enteric fever, FepE, inflammasomes, O‐antigen, pyroptosis, *Salmonella* Paratyphi A, *Salmonella* Typhimurium

## Abstract

*Salmonella* Paratyphi A (SPtA) remains one of the leading causes of enteric (typhoid) fever. Yet, despite the recent increased rate of isolation from patients in Asia, our understanding of its pathogenesis is incomplete. Here we investigated inflammasome activation in human macrophages infected with SPtA. We found that SPtA induces GSDMD‐mediated pyroptosis via activation of caspase‐1, caspase‐4 and caspase‐8. Although we observed no cell death in the absence of a functional *Salmonella* pathogenicity island‐1 (SPI‐1) injectisome, HilA‐mediated overexpression of the SPI‐1 regulon enhances pyroptosis. SPtA expresses FepE, an LPS O‐antigen length regulator, which induces the production of very long O‐antigen chains. Using a Δ*fepE* mutant we established that the very long O‐antigen chains interfere with bacterial interactions with epithelial cells and impair inflammasome‐mediated macrophage cell death. *Salmonella* Typhimurium (STm) serovar has a lower FepE expression than SPtA, and triggers higher pyroptosis, conversely, increasing FepE expression in STm reduced pyroptosis. These results suggest that differential expression of FepE results in serovar‐specific inflammasome modulation, which mirrors the pro‐ and anti‐inflammatory strategies employed by STm and SPtA, respectively. Our studies point towards distinct mechanisms of virulence of SPtA, whereby it attenuates inflammasome‐mediated detection through the elaboration of very long LPS O‐polysaccharides.

## 1 INTRODUCTION

Enteric (typhoid) fever is a life‐threatening disease caused by the *Salmonella enterica* serovars Typhi (STy) and Paratyphi A (SPtA). Both pathogens are human‐restricted, transmitted faecal‐orally and are common in areas with poor sanitation in middle and low‐income countries (Baker, Holt, et al., 2011; Coburn, Grassl, & Finlay, 2007). There are 11–20 million estimated enteric fever cases annually, with >200,000 associated deaths (Stanaway et al., 2019). The rate of SPtA isolation has been on the rise in recent years, and it is currently suspected that >40% of enteric fever cases in India, Pakistan and Nepal are caused by SPtA (Zellweger et al., 2017; Zhou et al., 2014).

SPtA and STy have independently evolved mechanisms to disseminate systemically and persist in secondary organs (Hiyoshi, Tiffany, Bronner, & Baumler, 2018; Holt et al., 2009). Despite the high degree of genetic relatedness and the plethora of shared pseudogenes, it remains unclear what shared or distinct genomic elements and virulence factors are responsible for enteric fever caused by these serovars (Hiyoshi, Tiffany, et al., 2018; Holt et al., 2009; Johnson, Mylona, & Frankel, 2018; McClelland et al., 2004). For example, the Vi capsule, which is present in STy, but absent from SPtA, has been suggested to mediate immune evasion (Hiyoshi et al., 2018; Wilson et al., 2008; Winter, Winter, Poon, et al., 2014; Winter, Winter, Atluri, et al., 2015). Alternatively, immune evasion by SPtA is thought to be mediated by the production of very long LPS O‐antigen chains, a process driven by the polysaccharide copolymerase FepE, which is a pseudogene in STy (Hiyoshi, Wangdi, et al., 2018). Both the Vi capsule and very long O‐chains promote the avoidance of the respiratory burst from neutrophils (Hiyoshi, Wangdi, et al., 2018).

Central to *Salmonella* virulence are two type III secretion systems (T3SS), encoded on *Salmonella* pathogenicity island 1 (SPI‐1) and SPI‐2, which secrete effectors that subvert host cell processes (Jennings, Thurston, & Holden, 2017). SPI‐1 T3SS mediates invasion into non‐phagocytic host cells and is readily recognised by innate immune pathways in macrophages (Srikanth, Mercado‐Lubo, Hallstrom, & McCormick, 2011; Wemyss & Pearson, 2019). Following internalisation, *Salmonella* resides within the *Salmonella‐*containing vacuole (SCV), which is maintained by the activity of the SPI‐2 effectors (Jennings et al., 2017). Although the molecular pathogenesis and virulence of SPtA are poorly understood, they are assumed to be similar to those of other *Salmonella* serovars like STy and the well‐studied non‐typhoidal *Salmonella* (NTS) serovar Typhimurium (STm). The latter has frequently been used as a model for typhoidal *Salmonella*, despite typhoidal and NTS resulting in different disease symptoms in humans, possessing different virulence genes and regulating essential virulence factors in a disparate manner (Gal‐Mor, Boyle, & Grassl, 2014; Johnson, Mylona, & Frankel, 2018; McDowell et al., 2019; Sabbagh, Forest, Lepage, Leclerc, & Daigle, 2010).

Macrophages play an important role during infection and systemic dissemination of *Salmonella* (Dougan & Baker, 2014). They can promote host defence by sensing and responding to infection via inflammasomes, which are multimeric complexes serving as cytosolic caspase‐1 activation platforms. Inflammasome activation is initiated upon recognition of bacterial conserved patterns by host proteins such as NLRs (NOD and leucine‐rich repeat containing proteins), PYRIN and AIM2. Caspase‐1 activation triggers pyroptotic cell death and proteolytic processing and secretion of pro‐inflammatory cytokines IL‐1β and IL‐18, resulting in an inflammatory response (Sanchez‐Garrido, Slater, Clements, Shenoy, & Frankel, 2020). Gasdermin‐D (GSDMD) cleavage liberates its N‐terminal region which forms pores in the cell membranes and triggers cell death by pyroptosis (Ding et al., 2016; Liu et al., 2016). Pyroptosis can also be induced via the non‐canonical pathway through cleavage of GSDMD by caspase‐4, which binds to, and is activated by, cytosolic LPS (Casson et al., 2015; Kayagaki, Stowe, et al., 2015; Shi, Zhao, Wang, Gao, et al., 2014; Shi, Zhao, Wang, Shi, et al., 2015). Innate immune signalling, proinflammatory cytokines or interferons (signal 1) regulate the expression and activity of inflammasome components (e.g., NLRP3) and promote bacterial recognition through a second signal that activates inflammasomes (signal 2) (Sanchez‐Garrido et al., 2020).

Most of our understanding of the roles inflammasomes play in *Salmonella* pathogenesis is based on studies of the interaction of STm with mouse macrophages (reviewed in [Sanchez‐Garrido et al., 2020; Wemyss & Pearson, 2019]). In macrophages, STm infection can be detected by caspase‐11 (human caspase‐4) and sensed by NLRP3, which recruits the adaptor ASC (apoptosis‐associated speck‐like protein containing a CARD) that oligomerises into “specks” within which caspase‐1 is activated (Broz et al., 2012; Casson et al., 2015; Fisch, Bando, et al., 2019). The single NAIP (neuronal apoptosis inhibitor protein) gene in humans, which corresponds to multiple mouse *Naip* genes, detects STm flagellin and the SPI‐1 T3SS rod (PrgJ) and needle (PrgI) proteins, leading to NLRC4 inflammasome activation (Kortmann, Brubaker, & Monack, 2015; Reyes Ruiz et al., 2017; Yang, Zhao, Shi, & Shao, 2013). Caspase‐8 also plays a role in STm detection as it regulates NLRP3 expression, and can be recruited to ASC specks (Gurung & Kanneganti, 2015; Man, Tourlomousis, et al., 2013; Man, Ekpenyong, et al., 2014). Despite their clinical importance, little is currently known about the function of inflammasomes during infection with typhoidal *Salmonella*.

The aim of this study was to determine if and how SPtA activates inflammasomes in human macrophages. We show that the SPI‐1 T3SS of SPtA is required for inflammasome‐dependent cell death via activation of caspase‐1 and caspase‐4. Conversely, we found that, by elaborating very long surface O‐antigen chains, SPtA dampens pyroptotic cell death. Taken together our data show that SPtA employs a novel stealth infection strategy.

## 2 RESULTS

### 2.1 *S*. Paratyphi A induces cell death in human macrophages

**FIGURE 1:**
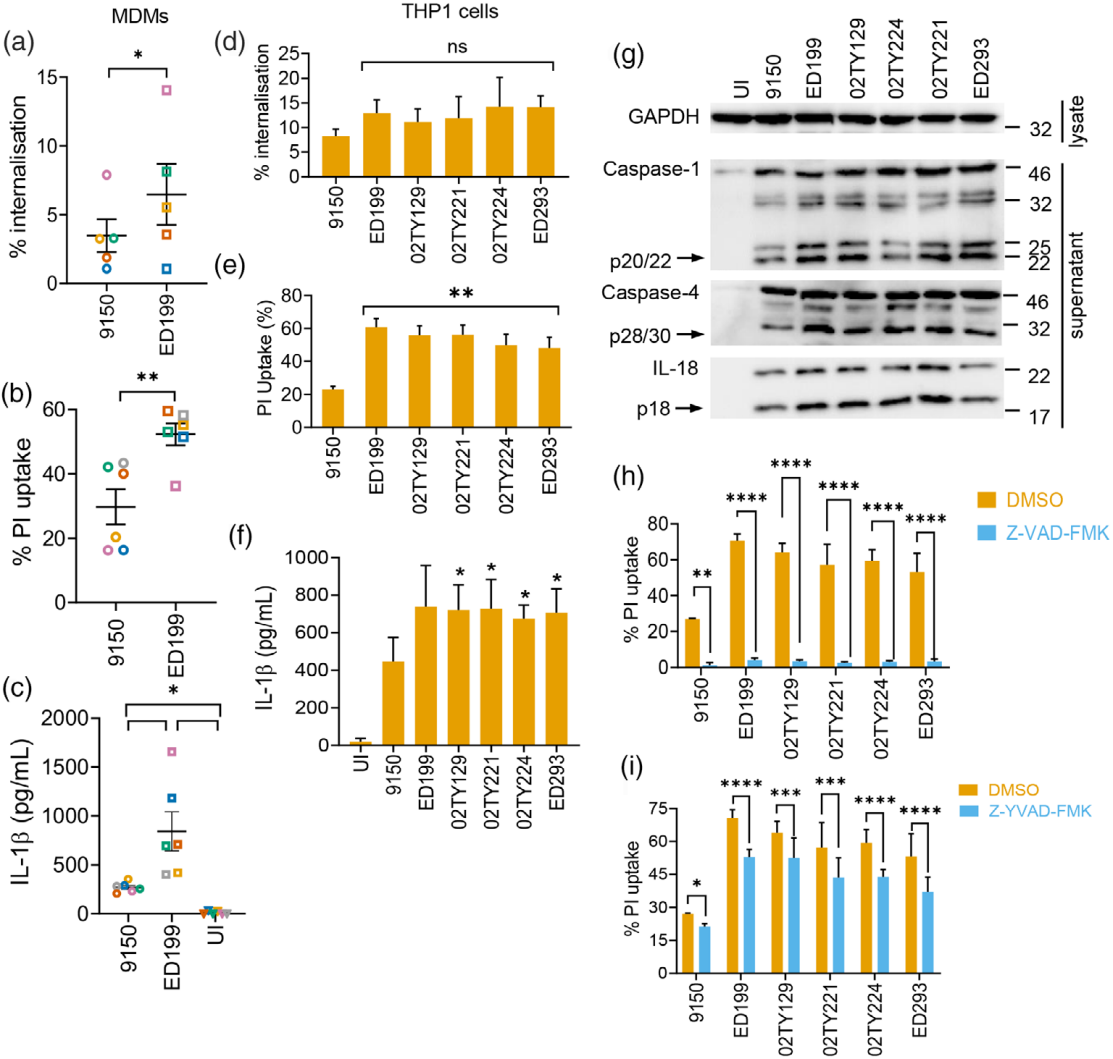
SPtA triggers caspase‐dependent cell death in human macrophages. (a) Bacterial internalisation (at 1.5 h post‐infection) and (b) PI uptake into MDMs infected with SPtA ED199 or 9150 (at 3 h post‐infection). (c) ELISA quantification of IL‐1β in supernatants of MDMs infected with the indicated SPtA strains (at 3 h post‐infection). (d) Bacterial internalisation and (e) PI uptake into THP1 cells infected with the indicated SPtA strains. (f) ELISA quantification of IL‐1β in THP1 cell supernatants infected with the indicated SPtA isolates. (g) Representative immunoblots (of two biological repeats) of SPtA‐infected THP1 cells showing cleaved products of caspase‐1, caspase‐4 and IL‐18 (arrows). (h, i) PI uptake into SPtA‐infected THP1 cells treated with Z‐VAD‐FMK (h) or Z‐YVAD‐FMK (i). Mean ± SEM from six independent donors in (a–c), 3 (d, f), 5 (e), or 6 (h, i) independent experiments are depicted and were compared by paired Student's *t*‐test (a, b), matched one‐way ANOVA (c–f) or two‐way ANOVA (h, i). In (d–f) SPtA 9150 was compared to all other strains. * *p* < *.05*, ** *p* < *.01*, *** p < *.001*, **** p < *.0001* after correction for multiple comparisons; ns, not significant

We infected primary human monocyte‐derived macrophages (MDMs) with the reference SPtA strain ATCC 9150 (SPtA 9150) and a clinical isolate originated from a patient with enteric fever in Nepal, SPtA ED199. At 3 h post infection, SPtA ED199 was internalised at significantly higher levels than SPtA 9150 (Figure 1a). Consistently, compared to SPtA 9150, SPtA ED199 induced two‐fold higher cell death, as measured by propidium iodide (PI) uptake (Figure 1b), and about three‐fold higher secretion of IL‐1β (Figure 1c).

We next assessed whether these phenotypes are also observed in differentiated human macrophage‐like THP1 cells, and furthermore tested four additional Nepalese clinical isolates (Table S1). The four clinical isolates were internalised similarly to SPtA ED199 and triggered equivalent cell death and secretion of IL‐1β (Figure 1d–f). Mechanistically, the different SPtA strains induced proteolytic activation of caspase‐1, caspase‐4 and IL‐18, with the clinical isolates showing higher potency in comparison to SPtA 9150 (Figure 1g), which correlated with their ability to induce greater PI uptake.

To assess the role of caspases in SPtA‐induced cell death, we pre‐treated THP1 cells with the pan‐caspase inhibitor Z‐VAD‐FMK, which effectively blocked nigericin‐induced pyroptosis as a control (Figure S1A). Z‐VAD‐FMK treatment abolished cell death during infection with SPtA 9150 and the five clinical isolates (Figure 1h). To determine if the necrotic cell death seen upon SPtA infection is mediated by inflammatory caspases, we pre‐treated THP1 cells with the narrow‐spectrum inhibitor Z‐YVAD‐FMK, which specifically inhibits caspase‐1/4. In control experiments Z‐YVAD‐FMK treatment reduced PI uptake induced by LPS transfection (Figure S1B). Importantly, Z‐YVAD‐FMK significantly reduced PI uptake induced by SPtA (Figure 1i), but not to the levels seen with Z‐VAD‐FMK, pointing towards the involvement of other caspases in SPtA‐induced cell death. We therefore additionally pre‐treated THP1 cells with the caspase‐8 inhibitor Z‐IETD‐FMK, which reduced staurosporine‐induced toxicity in control experiments (Figure S1C). Caspase‐8 inhibition also reduced cell death triggered by all the SPtA strains tested (Figure S1D). Collectively, these results implicate caspase‐1, caspase‐4 and caspase‐8 in SPtA‐induced cell death.

### 2.2 *S*. Paratyphi A induces pyroptotic cell death

**FIGURE 2:**
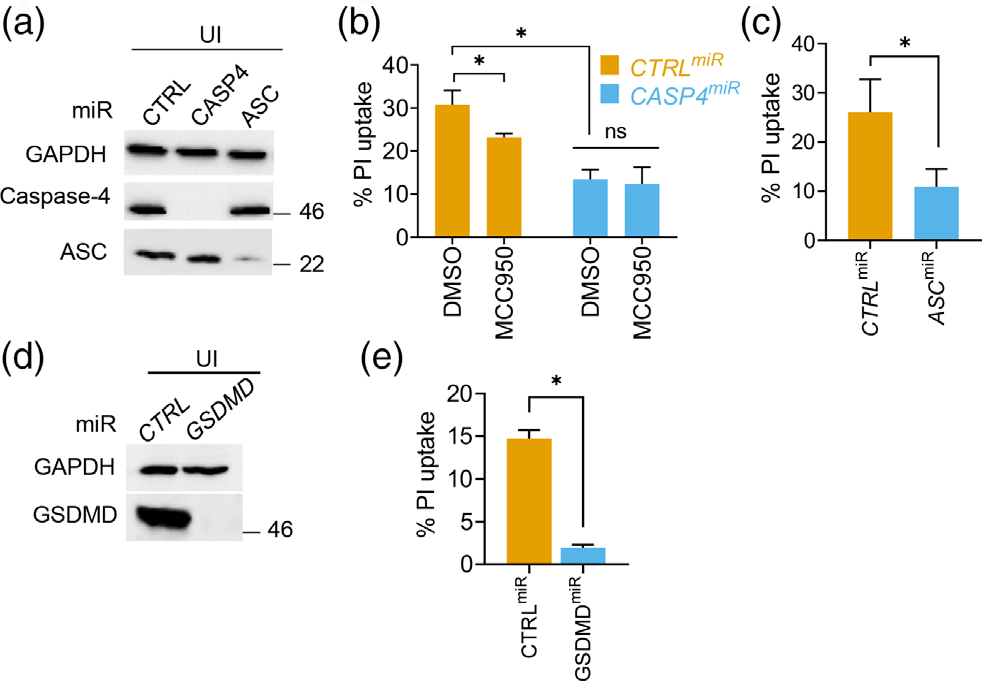
SPtA 9150 engages caspase‐4, ASC and the NLRP3 inflammasome to induce pyroptosis. (a) Representative immunoblots from THP1 cell lysates stably expressing non‐targeting (CTRL) or miRNA30E (miR) against caspase‐4 or ASC as labelled. (b) PI uptake into THP1 cells treated with vehicle (DMSO) or MCC950 and infected with SPtA 9150. (c) PI uptake into CTRL^miR^ or ASC^miR^ THP1 cells infected with SPtA 9150. (d) Immunoblots in THP1 cell lysates stably transduced with miRNA30E (miR) against *GSDMD* or a non‐targeting control (*CTRL*) as indicated. (e) PI uptake into the indicated THP1 cells infected with SPtA 9150. Mean ± SEM from 4 (b, c), or 3 (e) independent experiments are shown. * *p* < *.05* by paired Student's *t*‐test (c–e) or the indicated comparisons by two‐way ANOVA (B; with correction for multiple comparisons); ns, not significant

To validate the involvement of caspase‐4 and caspase‐1 in SPtA‐induced cell death we stably silenced expression of caspase‐4 (*CASP4* ^miR^) and the caspase‐1 adaptor ASC (*ASC* ^miR^) in THP1 cells (Figure 2a); we also used the NLRP3‐specific inhibitor MCC950 (Coll et al., 2015). Successful silencing or inhibition was functionally confirmed using LPS transfection (an activator of caspase‐4) and LPS priming followed by nigericin (NLRP3/ASC activation) (Figures S1E and S2A,B). Infection with SPtA 9150 revealed that silencing of caspase‐4 or ASC reduced pyroptosis by ~40% (Figure 2b,c) and MCC950 reduced PI uptake by ~25% (Figure 2b), pointing towards other ASC‐dependent inflammasomes being involved in SPtA detection. Combined inhibition of NLRP3 and caspase‐8 with MCC950 and Z‐IETD‐FMK led to a ~75% reduction in cell death; however, these inhibitors did not further reduce pyroptosis in *CASP4* ^miR^ cells (Figure 2b and Figure S2C). While ASC is required for caspase‐1 activation and IL‐18 processing, caspase‐4 silencing did not affect caspase‐1 activation (Figure S2D). This is consistent with PI uptake assays (Figure 2b) and suggested that caspase‐1 could be activated in the absence of caspase‐4. Collectively, these data suggest that activation of caspase‐4 by SPtA directly induces pyroptosis, while it also drives the NLRP3‐ASC‐caspase‐1 non‐canonical pathway. Additional redundant mechanisms of pyroptosis involved caspase‐8 and ASC‐dependent activation of caspase‐1 by as yet unidentified sensors (Gurung et al., 2014; Man, Tourlomousis, et al., 2013; Man, Ekpenyong, et al., 2014; Sarhan et al., 2018).

As caspase‐1 and caspase‐4 execute pyroptosis via proteolysis of GSDMD, we tested its involvement in SPtA‐induced pyroptosis. To this end, we knocked down *GSDMD* expression in THP1 cells using a miRNA30E‐based plasmid (*GSDMD* ^*miR*^) (Figure 2d), which was functionally validated by the reduction in pyroptosis after nigericin‐triggered canonical NLRP3 inflammasome activation (Figure S2E). *GSDMD* silencing prevented SPtA‐induced cell death as compared to CTRL^miR^ cells (Figure 2e). Taken together, caspase‐1, caspase‐4, caspase‐8, NLRP3 and ASC‐dependent inflammasome‐mediated sensing of SPtA contributes to macrophage cell death through pyroptosis.

### 2.3 The SPtA 9150 SPI‐1 T3SS triggers pyroptosis

**FIGURE 3:**
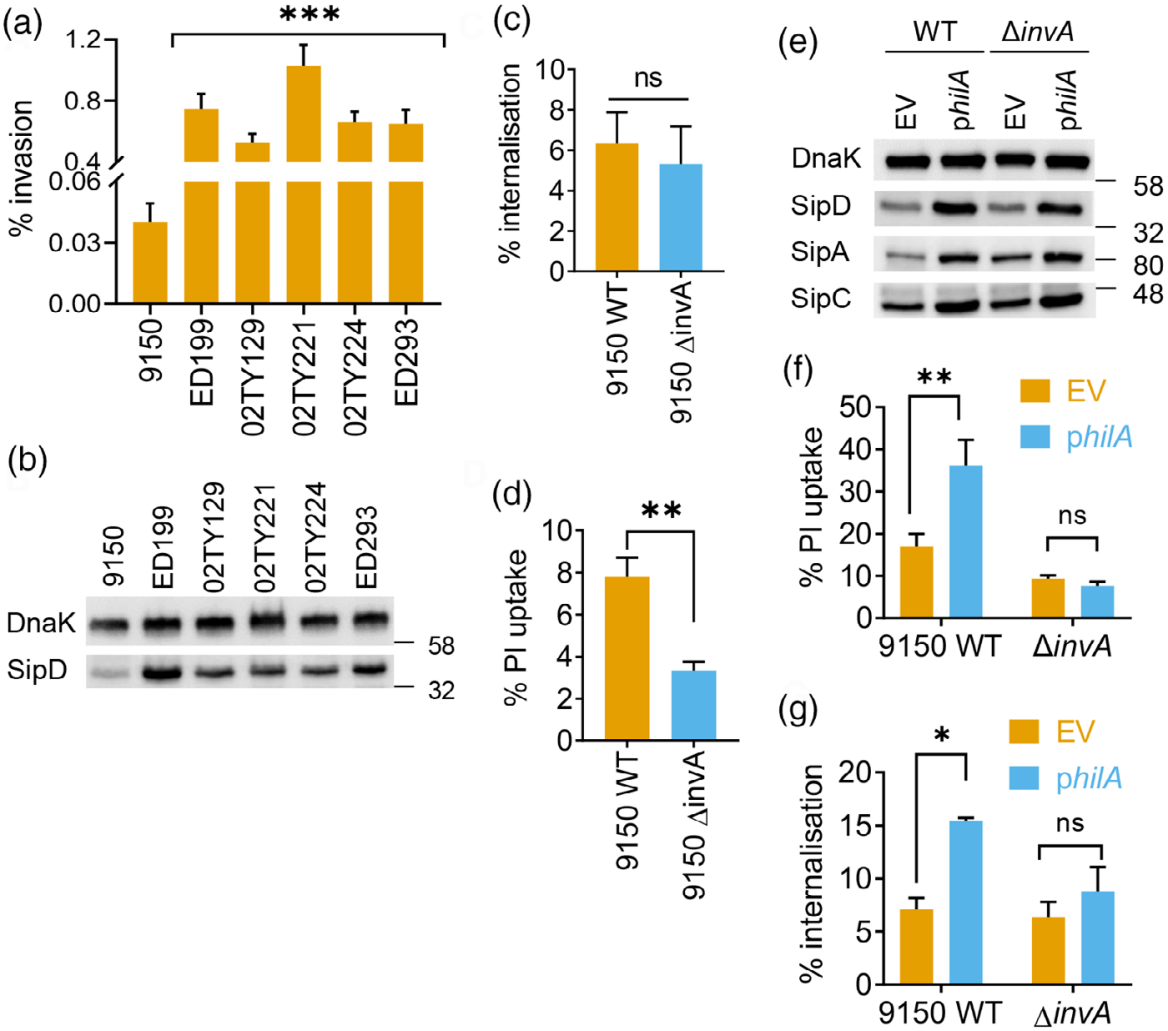
SPtA SPI‐1 T3SS is required for macrophage pyroptosis. (a) Invasion of HeLa cells by the indicated SPtA strains. (b) Representative (of three biological repeats) immunoblots for the SPI‐1 effector SipD and DnaK (loading control) in lysates of the indicated SPtA isolates. (c) Bacterial internalisation and (d) PI uptake into THP1 cells infected with the indicated SPtA 9150 strains. (e) Representative immunoblots (of two biological repeats) for the indicated SPI1‐associated proteins or DnaK (loading control) in the indicated strains of SPtA with empty vector (EV) or HilA expression plasmid. (f) PI uptake and (g) bacterial internalisation by THP1 cells infected with the indicated SPtA 9150 strains. Mean ± SEM from 5 (a, c), 6 (d), or 3 (f, g) independent experiments. * *p* < *.05*, ** *p* < *.01*, *** *p* < *.001* for the indicated comparisons by Student's *t*‐test (c, d), one‐way (a) or two‐way ANOVA (f, g) following correction for multiple comparisons; ns, not significant

We aimed to identify SPtA‐encoded virulence factors that could induce pyroptotic cell death, starting with the SPI‐1‐encoded T3SS. We first investigated the activity and expression of the SPI‐1 T3SS in SPtA. As SPtA is internalised by macrophages through phagocytosis independently of the T3SS, we infected HeLa cells with SPtA 9150 and the five clinical isolates; invasion was assessed by gentamicin‐protected intracellular colony forming unit (CFU) enumeration after 2 h. The five clinical SPtA isolates invaded HeLa cells at a markedly higher percentage than SPtA 9150 (Figure 3a), which correlated with a higher expression of a representative SPI‐1 T3SS effector, SipD (Figure 3b).

We next deleted *invA* in SPtA 9150, which prevents secretion of effectors via the SPI‐1 T3SS (Elhadad et al., 2016). As expected, SPtA 9150 Δ*invA* did not invade HeLa cells (Figure S3A). In addition, while wildtype (WT) SPtA 9150 and Δ*invA* were internalised similarly by THP1 cells (Figure 3c), SPtA 9150 Δ*invA* induced ~2‐fold lesser cell death in THP1 cells than the parental WT strain (Figure 3d). In a reciprocal experiment, we introduced the global positive regulator of SPI‐1 genes, HilA, expressed from a constitutive T3 promoter, into WT SPtA 9150; overexpression of HilA in Δ*invA* served as a negative control. Overexpression of HilA increased expression of SPI‐1 genes in both SPtA 9150 and the Δ*invA* strains, as seen from immunoblots for SipA, SipC and SipD (Figure 3e), in line with previous findings (Elhadad et al., 2016); but only specifically increased the invasion of WT SPtA 9150, not Δ*invA*, into HeLa cells (Figure S3B). Overexpression of HilA also triggered higher pyroptosis of THP1 cells as compared to SPtA 9150 carrying the empty vector (EV), while it did not affect pyroptosis induced by the Δ*invA* strain (Figure 3f). HilA‐mediated increase in THP1 cell pyroptosis correlated with increased internalisation of WT, but not Δ*invA* bacteria (Figure 3g), suggesting that the upregulation of SPI‐1 enabled active invasion of macrophages by SPtA. Taken together, these results implicate the SPI‐1 T3SS in SPtA‐induced pyroptosis, but not uptake by macrophages.

As the STm flagellin and T3SS needle and rod can activate the NAIP/NLRC4 inflammasomes (Kortmann et al., 2015; Reyes Ruiz et al., 2017; Yang et al., 2013), we asked whether they play a role in mediating SPtA 9150‐induced pyroptosis. We generated *NAIP* ^*miR*^ THP1 cells, which showed an effective reduction of NAIP protein levels (Figure S3C). Surprisingly, neither NAIP silencing nor further inhibiting NLRP3 with MCC950 treatment affected caspase‐1 activation (Figure S3C) or pyroptosis (Figure S3D) during infection with SPtA 9150. This suggested that the NAIP pathway plays a minor role in detecting SPtA in this experimental scenario.

### 2.4 Very long O‐antigen chains interfere with inflammasome activation

**FIGURE 4:**
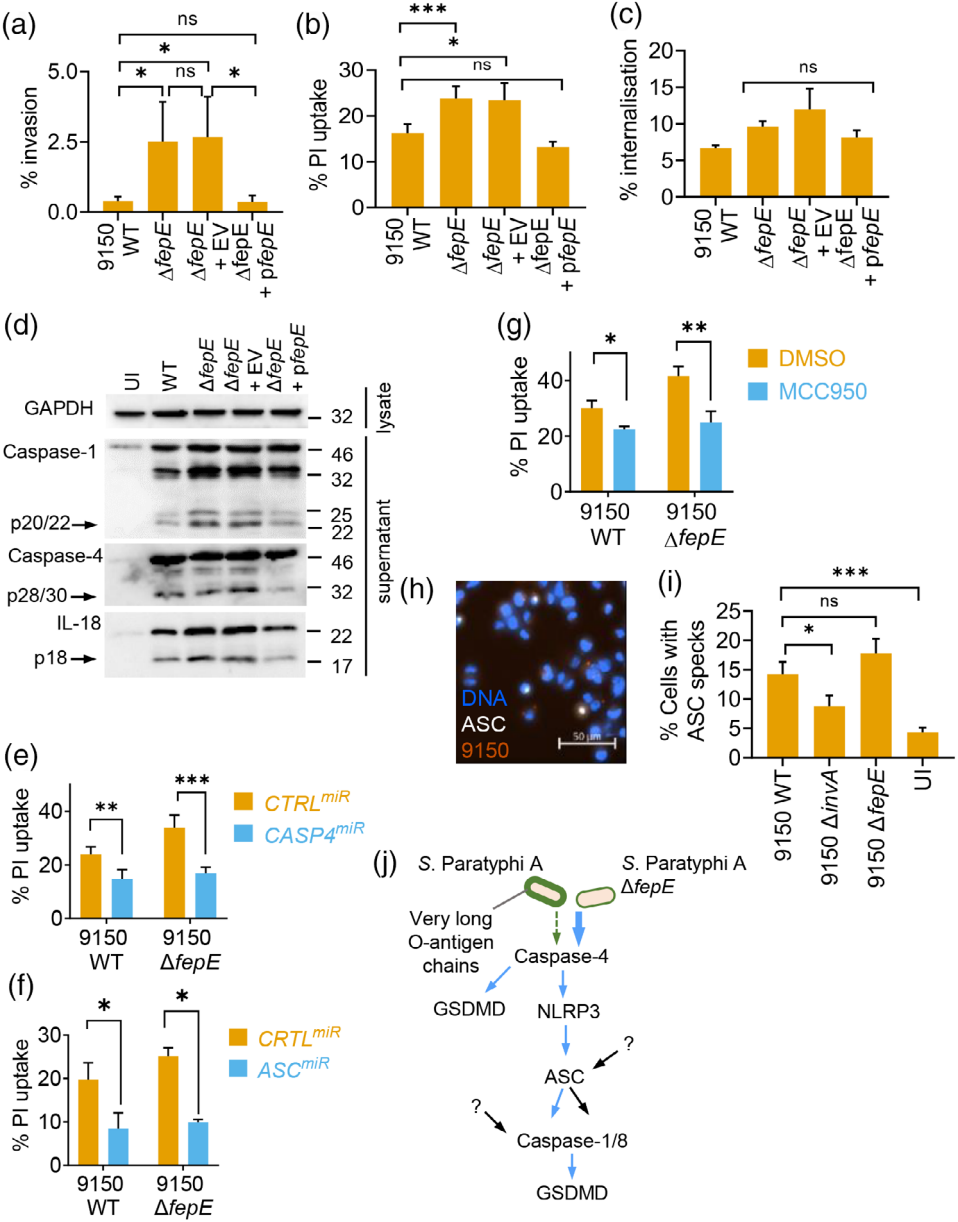
Very long LPS O‐antigen chains interfere with inflammasome activation by SPtA. (a) Invasion of HeLa cells by WT SPtA 9150, Δ*fepE* or Δ*fepE* complemented with an EV or a FepE‐encoding plasmid (p*fepE*). (b) PI uptake and (c) bacterial internalisation into THP1 cells infected with the indicated SPtA 9150 strains. (d) Representative immunoblots (of 3 biological repeats) from cell lysates or supernatants of THP1 cells infected with the indicated SPtA 9150 strains showing cleaved caspase‐1, caspase‐4 and IL‐18 (arrows). (e, f) PI uptake into THP1 cells expressing non‐targeting control (CTRL), CASP4 or ASC‐targeting miRNA30E (miR) infected with the indicated strains of SPtA. (g) PI uptake into DMSO or MCC950‐treated THP1 cells infected with the indicated SPtA 9150 strains. (h, i) Representative fluorescence microscopy image (h) and quantification (i) of ASC specks in THP1‐ASC^mRFP^ cells infected with WT SPtA 9150 (h) or the indicated strains (i). (j) Proposed model of inflammasome activation in human macrophage infected with SPtA. Mean ± SEM from 3 (a, c, f), 4 (b, i), or 5 (e, g) independent experiments. * *p* < *.05*, ** *p* < *.01*, *** *p* < *.001* for indicated comparisons by one‐way (a–c, i), two‐way (e–g ANOVA after correction for multiple comparisons; ns, not significant

The T3SSs in *Shigella* and enterohemorrhagic *E. coli* (EHEC) have been shown to be masked and inhibited by the LPS O‐antigen and group 4 capsule, respectively (Caboni et al., 2015; Shifrin et al., 2008; Watson et al., 2019b; West et al., 2005). As FepE mediates the production of very long LPS O‐antigen chains in SPtA, we hypothesised that these may interfere with the function of the T3SS and pyroptosis. To test this, we generated a SPtA 9150 Δ*fepE* mutant, which reduced the production of very long polymers of O‐antigen polysaccharides, without affecting bacterial growth in standard laboratory growth media (Figure S4A,B). SPtA 9150 Δ*fepE* was ~6‐fold more invasive into HeLa cells than the parental WT strain (Figure 4a). Complementation of SPtA 9150 Δ*fepE* with *fepE*, constitutively expressed on a low‐copy number plasmid (pWSK29‐*fepE*), restored the expression of very long O‐antigen polysaccharides (Figure S4B) and invasion into HeLa cells to levels seen with the WT strain (Figure 4a). These results suggest that the very long O‐antigen chains may mask the activity of the SPI‐1 T3SS in SPtA.

We next investigated the effect of very long LPS O‐antigen chains on pyroptosis. SPtA 9150 Δ*fepE* triggered higher cell death compared to WT, which was reduced to levels comparable to WT upon *fepE* complementation (Figure 4b). SPtA 9150 WT, Δ*fepE* and the complemented strain were phagocytosed similarly (Figure 4c), suggesting that differences in cell death were not due to differential bacterial internalisation by macrophages. LPS is a dominant molecule from Gram‐negative bacteria that provides signal 1 for priming NLRP3 inflammasome components. We therefore tested whether Δ*fepE* bacteria, which have altered LPS O‐antigen chains, induced more pro‐inflammatory signalling. Infection with SPtA 9150 WT and Δ*fepE*, as well as Δ*invA* as a control, induced similar secretion levels of TNF, which is induced and secreted via inflammasome‐independent signalling (Figure S4C), ruling out a role for the LPS O‐antigen chains in macrophage priming. To further validate this conclusion, we primed THP1 cells by treating them with *E. coli* O111:B5 LPS; despite similar priming, cell death caused by SPtA 9150 Δ*fepE* remained higher than that induced by WT (Figure S4D). Immunoblotting of caspase‐1, caspase‐4 and IL‐18 revealed that deletion of *fepE* led to an increasing trend of activation of these proteins as compared to WT SPtA 9150 and the complemented strain (Figure 4d and Figure S4E‐G). Furthermore, like WT SPtA 9150 (Figure 2b,c), pyroptosis by Δ*fepE* was also reduced by silencing caspase‐4 or ASC or inhibition of NLRP3 and caspase‐8 (Figure 4e–g and Figure S4H). These results established that SPtA lacking very long LPS O‐antigen chains triggered enhanced inflammasome activation compared to WT.

Inflammasomes are cytosolic multiprotein complexes and thus require the bacteria or their molecules to be present in the cytosol to be recognised. To examine whether SPtA escapes the SCV, we quantified cytosolic bacteria by chloroquine‐resistance assays in the pyroptosis‐resistant *GSDMD* ^miR^ THP1 cells. At 1.5 h post infection, chloroquine‐resistant cytosolic SPtA 9150 were detected at low numbers, and bacterial escape into the cytosol was independent of SPI‐1 T3SS or the very long O‐antigen chains (Figure S5A). However, at 3 h post infection, there were fewer Δ*invA* mutant bacteria in the cytosol as compared to SPtA 9150 WT or Δ*fepE*, suggesting that vacuolar escape later during infection is dependent on the SPI‐1 T3SS but is not affected by the very long O‐antigen chains (Figure S5B). These results suggest that basal T3SS activity of WT SPtA is sufficient to mediate rupture of the SCV after bacterial phagocytosis by macrophages.

To further verify the dominant role of caspase‐4 in detecting cytosolic Δ*fepE* bacteria, we quantified ASC assembly into inflammasome specks by using a THP1 cell line expressing RFP‐tagged ASC (ASC^mRFP^) (Figure 4h). WT and Δ*fepE* bacteria induced ASC‐speck formation to comparable levels (Figure 4i and Figure S5C). We therefore concluded that increased caspase‐4 activation by SPtA Δ*fepE* results in elevated pyroptosis directly, rather than through downstream activation of NLRP3‐ASC‐caspase‐1 inflammasomes, while additional ASC‐dependent pathways are also expected to be involved (Figure 4j). Taken together, these results suggest that the very long LPS O‐chains limit inflammasome activation and pyroptosis after bacterial escape from vacuoles, which represents a unique mechanism of suppressing caspase‐4 activation.

### 2.5 FepE expression inversely correlates with *Salmonella*‐induced pyroptosis

**FIGURE 5:**
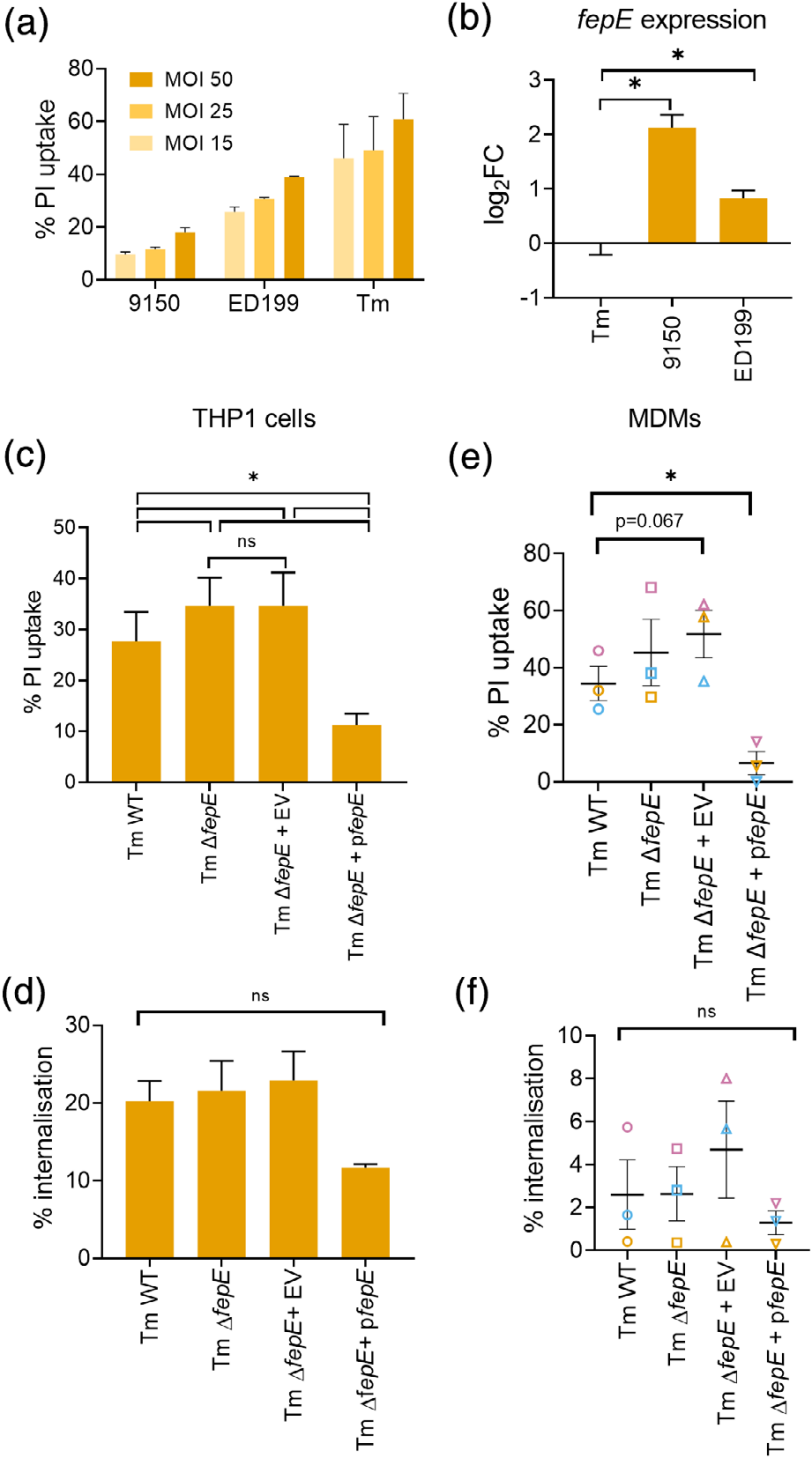
*fepE* expression inversely correlates with cell death induction by *Salmonella*. (a) PI uptake into THP1 cells infected with SPtA 9150, ED199 or STm at the indicated MOIs. (b) *fepE* expression in the indicated strains as assessed by qPCR. Log2 fold change is shown compared to STm expression. (c–f) PI uptake (c, e) and bacterial internalisation (d, d) into THP1 cells or MDMs, respectively, infected with WT STm, Δ*fepE* or Δ*fepE* complemented with an EV or a FepE‐encoding plasmid (p*fepE*). Mean ± SEM from 2 (a), 4 (b), 3 (c–f) independent experiments. * *p* < *.05* for indicated comparisons by one‐way ANOVA following correction for multiple comparisons; ns, not significant

Although *fepE* is a pseudogene in STy, it is intact in STm and highly similar to that of SPtA 9150 (Figure S6). We infected THP1 cells at increasing MOIs with SPtA 9150, ED199 and STm and observed that, across all MOIs, SPtA 9150 induced the lowest levels of pyroptosis and STm the highest (Figure 5a). Therefore, given that FepE‐mediated production of very long O‐antigen chains prevented pyroptosis (Figure 4b), we hypothesised that SPtA 9150 may be eliciting a low inflammasome signalling response due to high expression of FepE. To test this, we performed RT‐qPCR to determine *fepE* expression in SPtA 9150 and ED199 compared to STm. This revealed that SPtA 9150 expressed *fepE* at much higher levels than STm and SPtA ED199, which expressed *fepE* at an intermediate level (Figure 5b).

We next investigated whether very long O‐antigen chains in STm play a role in dampening cell death in human macrophages. Deletion of *fepE* resulted in a small, but significant increase in cell death triggered by STm, which was restored to WT levels upon complementation with plasmid‐borne *fepE* (Figure 5c); all the strains were similarly internalised by THP1 cells (Figure 5d). A similar phenotype was observed upon infection of MDMs with STm (Figure 5e,f). Interestingly, the complemented STm Δ*fepE* strain induced markedly lower pyroptosis, which correlated with lower bacterial internalisation (Figure 5f). These results suggest that *fepE* expression inversely correlates with inflammasome activation and pyroptosis induced by *Salmonella* independently of the serovar.

## 3 DISCUSSION

Here we showed that SPtA induces caspase‐ and GSDMD‐dependent pyroptotic cell death in human macrophages. Notably, using pharmacological and genetic silencing, we found that SPtA‐induced pyroptosis is predominately dependent on caspase‐4 activation, with additional contributions made by the NLRP3/caspase‐1 inflammasome, caspase‐8 and additional ASC‐dependent sensors. The very long LPS O‐antigen chains expressed by SPtA did not affect macrophage priming, bacterial uptake or escape from the SCV, but they reduced inflammasome activation and pyroptosis.

We have recently showed that infection of macrophages with enteropathogenic *Escherichia coli* (EPEC) triggers a cell death cascade in which activation of caspase‐4 by LPS leads to activation of the NLRP3/ASC/caspase‐1 inflammasome and pyroptosis (Goddard et al., 2019), and caspase‐4 is also involved in recognition of STm infection in IFNγ‐primed macrophages (Fisch, Bando, et al., 2019; Fisch, Clough, et al., 2020; Kutsch et al., 2020; Santos et al., 2020; Wandel et al., 2020). Moreover, we observed that caspase‐8, which has been shown to induce GSDMD cleavage (Orning et al., 2018; Sarhan et al., 2018), also contributed to cell death by SPtA. Alternatively, caspase‐8 may promote macrophage priming (Gurung et al., 2014; Van Opdenbosch et al., 2017). As stable caspase‐8 silencing or knockout of caspase‐8 is lethal in THP1 cells (data not shown), we could not test its roles further. Our genetic and pharmacological approaches also point towards a dominant role for ASC in cell death by SPtA, as its silencing led to comparable reduction in pyroptosis to caspase‐4 silencing. The partial effect of NLRP3 inhibition with MCC950 on pyroptosis suggests that as yet other inflammasome sensors may detect SPtA via ASC (Figure 4j). In addition, we ruled out a major role for NAIP‐NLRC4 inflammasomes in detecting SPtA. Future work should investigate the redundancy in SPtA‐sensing in human macrophages.

Similar to what is observed in STm (Bierschenk et al., 2019; Miao et al., 2006), STy (unpublished data) and EPEC infections (Goddard et al., 2019), the T3SS is required for cell death during infection with SPtA. Although SPI‐1 T3SS did not mediate initial bacterial uptake by macrophages, it facilitated escape to the cytosol, evidently promoting pathogen recognition by host cell intracellular sensors. Consistent with this, the higher level of SPI‐1 expression in clinical isolates correlated with their ability to cause more pyroptosis. Bacterial internalisation by macrophages was enhanced once SPI‐1 was overexpressed via HilA, suggesting that SPtA may limit SPI‐1 expression as an immune evasion mechanism. Suppression of pyroptosis by SPtA could be beneficial for the pathogen to either escape killing by extracellular mechanisms following cell death and release of the bacteria [such as pore‐induced intracellular traps (Jorgensen, Zhang, Krantz, & Miao, 2016)] and/or, more likely, to promote survival of the host cell and thus increase dissemination of the pathogen within macrophages to systemic sites. Reduced SPI‐1 expression could also underlie the lack of a role for NAIP‐NLRC4 inflammasomes in detecting SPtA even though its SPI‐1 NAIP ligands PrgI and PrgJ are ~96% and 100% identical, respectively, to those in STm. Further, the FliC proteins in SPtA and STm are only 76% identical suggesting that, like *E. coli* flagellin, it may not be detected by the NAIP receptor. Experiments with recombinant proteins are necessary to conclusively show whether SPtA flagellin can activate human NAIP‐NLRC4 pathways. Moreover, in addition to reduced SPI‐1 T3SS expression (through HilA regulation) or access of the SPI‐1 T3SS to immune sensors via very long O‐antigen chains (see below), it is plausible that a secreted effector in SPtA suppresses NAIP‐NLRC4 signalling.

Very long LPS O‐antigen chains are produced by the polysaccharide copolymerase FepE. Although the regulation of FepE expression levels during the infection cycle of SPtA remains unclear and warrants further investigation, we speculate that its levels are higher during intracellular stages as has been observed with STm that reside in the SCV (da Silva, Manieri, Herrera, Trent, & Moreira, 2018). LPS chain length limits the accessibility of STm (Hölzer, Schlumberger, Jäckel, & Hensel, 2009), *Shigella flexneri* (West et al., 2005) and *Shigella sonnei* (Caboni et al., 2015) T3SS injectisomes to the host lipid bilayer, while the group 4 capsule masks the T3SS in EHEC (Shifrin et al., 2008). Very long O‐antigen chains in SPtA have been shown to reduce antibody‐mediated recognition by the host and respiratory burst in neutrophils (Hiyoshi, Wangdi, et al., 2018), and are also important for STm virulence (Hölzer et al., 2009; Murray, Attridge, & Morona, 2003, 2005, 2006). Here we show that SPtA very long O‐antigen chains restricted host epithelial cell invasion and suppressed SPtA‐induced pyroptosis but not vacuolar escape. Therefore, the length of LPS sugar chains might sterically hinder caspase‐4‐mediated recognition of lipid‐A in the cytosol (Shi, Zhao, Wang, Gao, et al., 2014), which appears to act upstream of the major inflammasome pathways activated during SPtA infection (Figure 4j). Loss of very long O‐antigen chains rapidly led to increased caspase‐4 activation and pyroptosis, but did not increase the percentage of cells containing ASC foci. A host cell can only assemble a single inflammasome “speck,” and because WT SPtA can also trigger detectable inflammasome activation (e.g., through a caspase‐4‐independent canonical inflammasome pathway), our findings suggest that the loss of *fepE* triggers higher pyroptosis mainly through direct activation of caspase‐4, enhanced GSDMD proteolysis, and membrane damage. While STm encodes FepE, it expresses it at much lower levels than SPtA and seems not to exploit this as an immune evasion mechanism. Importantly, we found an inverse correlation between FepE expression and the level of pyroptosis. SPtA and STm express *fepE* at different levels possibly as a result of their infection strategies in humans. STm induces an extensive inflammatory response in the small intestine which promotes competition with resident microbiota (Stecher et al., 2007), disruption of the intestinal barrier, penetration to the submucosa and neutrophil infiltration (Tükel et al., 2006; Zhang et al., 2003). In contrast, higher expression of FepE in SPtA leads to very long O‐antigen chains, which act as an immune evasion mechanism in the intestine (Hiyoshi, Wangdi, et al., 2018), allowing its systemic dissemination. This is analogous to the Vi antigen‐mediated escape from immune recognition in *S*. Typhi infection (Hiyoshi, Tiffany, et al., 2018; Johnson, Mylona, & Frankel, 2018).

Bacterial modifications of LPS acylation, for example by *Helicobacter*, *Francisella* and *Yersinia*, have previously been reported to suppress caspase‐4/11 activation in human and mouse macrophages (Hagar, Powell, Aachoui, Ernst, & Miao, 2013; Kayagaki, Wong, et al., 2013; Lagrange et al., 2018; Shi, Zhao, Wang, Gao, et al., 2014). However, unlike mouse caspase‐11, human caspase‐4 can detect hypo‐acylated LPS from *Francisella*, which indicates species‐specific differences (Lagrange et al., 2018). Other mechanisms of subverting caspase‐4 detection in epithelial cells include T3SS effectors that inhibit it, such as OspC3 from *Shigella* and NleF from EPEC and EHEC (Kobayashi et al., 2013; Pallett et al., 2016). Conversely, host macrophages can detect LPS through caspase‐4 during infection by STm (P. J. Baker, Boucher, et al., 2015; Casson et al., 2015; Fisch, Bando, et al., 2019; Fisch, Clough, et al., 2020) and EPEC (Goddard et al., 2019). Here we found that the modification of LPS O‐antigen polysaccharide length, which is dispensable for caspase‐4 binding (Hagar et al., 2013; Kayagaki, Wong, et al., 2013), can prevent caspase‐4 activation and help bacteria evade detection in human cells. O‐antigen‐mediated evasion of caspase‐4 activation may also be used by *S. sonnei* (Watson et al., 2019a, 2019b). Whether other bacteria that produce very long O‐antigen chains, such as *S*. Dublin and *S*. Enteritidis (Murray et al., 2003), *Pseudomonas aeruginosa* (Kintz, Scarff, DiGiandomenico, & Goldberg, 2008) and *S. flexneri* (Morona, Daniels, & Van Den Bosch, 2003), may also avoid caspase‐4 activation in this manner should be investigated in the future. GBP1 is an interferon‐γ‐stimulated guanylate binding protein which assists in caspase‐4 activation by LPS from cytosolic STm (Fisch, Bando, et al., 2019; Fisch, Clough, et al., 2020; Kutsch et al., 2020; Santos et al., 2020; Wandel et al., 2020). Although induction of GBP1 expression is not an essential component for caspase‐4 activation, for example during infection with EPEC (Goddard et al., 2019), future work should also investigate whether GBP1 or other GBPs can overcome the reduced inflammasome activation by very long LPS O‐antigen chains.

Our results also revealed that clinical SPtA isolates were more virulent than the prototype SPtA 9150 strain in terms of SPI‐1 T3SS expression and activity, inflammatory responses and macrophage cytotoxicity. These results are consistent with data generated from an outbreak SPtA isolate, which was more invasive and motile than SPtA 9150 (Gal‐Mor, Suez, et al., 2012). It is likely that in vitro adaptation over time has attenuated the prototype strain SPtA 9150, in a manner comparable to STy (Johnson, Ravenhall, et al., 2018). Alternatively, these differences may be the result of polymorphism acquisition in the geographical region where these organisms were isolated, a phenomenon seen with differing STy genotypes (Frankel, Newton, Schoolnik, & Stocker, 1989; Wong et al., 2015). Of note, our data show that SPtA ED199, which has been used in clinical human challenge studies (Dobinson et al., 2017; McCullagh et al., 2015), has similar pathogenicity to the other clinical variants used in this study.

In summary, we show that SPtA induces inflammasome‐dependent cell death during infection of human macrophages. This process is dependent on the SPI‐1 T3SS and is limited by production of very long O‐antigen chains. We propose a model (Figure 4j) whereby SPtA leads to caspase‐4‐dependent pyroptosis and NLRP3‐ASC‐caspase‐1 and/or caspase‐8 activation, while other yet unidentified ASC and/or caspase‐1 pathways are likely to be involved. Thus, SPtA interacts with human immune cells through mechanisms distinct to other *Salmonella* serovars as exemplified by the extent to which FepE‐mediated immune evasion is exploited by SPtA compared to STm. Observations such as those outlined here are critical for understanding how SPtA and other invasive *Salmonella* promote infection and how we could circumvent bacterial virulence strategies. As we move into an era of mass STy vaccination, we need to quickly comprehend common and distinct mechanisms of virulence given the increasing rates of SPtA infections.

## 4 EXPERIMENTAL PROCEDURES

### 4.1 Ethics statement

MDM cells were isolated from blood obtained from anonymous adult male and female donors to the NHS Blood and Transplant, Colindale, London. Experiments were performed in compliance and approval from the Imperial College Healthcare Tissue Bank.

### 4.2 Preparation of primary monocyte derived macrophages (MDMs)

Primary MDMs were prepared as previously described via CD14+ enrichment by MACS (Magnetic‐activated cell sorting, Miltenyi Biotec) (Goddard et al., 2019). CD14+ cells were confirmed by flow cytometry (85–95%).

### 4.3 Bacterial strains

Bacterial strains used in this study are listed in Table S1. *Salmonella* were routinely grown in Lysogeny Broth (LB) Lennox at 37°C with shaking at 200 rpm, with the addition of appropriate antibiotics where necessary (kanamycin (50 μg/ml) and spectinomycin (100 μg/ml); see also Table S4). SPI‐1 expression was induced by sub‐culturing following 1:33 dilution and growth to late exponential phase.

### 4.4 Generation of bacterial strains and plasmids

Deletion mutants were constructed using the λ‐red recombinase system as previously described (Datsenko & Wanner, 2000). Gene deletions were confirmed by sequencing (Eurofins/GATC). pWSK29‐Spec (Johnson, Byrne, et al., 2017) vectors were assembled by Gibson assembly according to manufacturer's instructions. Plasmid inserts were confirmed by sequencing with standard M13 primers (Eurofins/GATC). Primers are listed in Table S2.

### 4.5 Mammalian cell culture

All cell lines (Table S3) were maintained at 37°C and 5% CO_2_, and were confirmed to be mycoplasma‐negative (MycoAlert mycoplasma detection kit; Table S4). THP1 cells were routinely cultured in suspension in RPMI 1,640 supplemented with 10% heat‐inactivated foetal bovine serum (FBS), 1 mM Sodium Pyruvate, 10 mM HEPES solution, and 100 units/100 μg/mL penicillin/streptomycin (Table S4). Retroviral plasmid transduced cell lines were generated as previously described (Eldridge, Sanchez‐Garrido, Hoben, Goddard, & Shenoy, 2017) and additionally cultured with puromycin (2 μg/ml). HeLa cells were routinely maintained in adhesive flasks in DMEM 4,500 mg/ml glucose media supplemented with 10% heat‐inactivated FBS and 1% Glutamax (Table S4).

### 4.6 Cell treatments and in vitro infection

HeLa cells were seeded at 7 × 10^4^ cells/well 24 h prior to infection in 24‐well plates. THP1 cells were seeded in 96‐well black‐wall clear‐bottom plates at a density of 1.5 × 10^5^ cells/well for cell death assays, in 48‐well plates at 4.5 × 10^5^ cells/well for western blotting, or on glass coverslips in 24‐well plates at 5 × 10^5^ cells/well for immunofluorescence. Differentiation of THP1 cells to macrophage‐like cells was induced with 100 ng/mL phorbol 12‐myristate 13‐acetate (PMA; Table S4) for 48 h, before replacing media with complete media without antibiotics 24 h prior to infection or cell treatments. For cell death assays, cell treatments and infections were carried out in complete RPMI media lacking phenol red.

Where indicated, THP1 cells were primed with 250 ng/ml Ultra‐pure O111:B4 LPS‐EB for 3 h prior to treatment with 25 μM nigericin for 1 h. THP1 cells were treated with chemical inhibitors at 1 h prior to infection: Z‐VAD‐FMK (50 μM), Z‐YVAD‐FMK (50 μM), Z‐IETD‐FMK (50 μM) and MCC950 (2 μM). Caspase‐4 activation was induced in unprimed cells by transfecting LPS (5 μg/ml) with Lipofectamine 2,000 (1% v/v) for 3 h. Caspase‐8 activation was induced by treating unprimed cells with staurosporine (2 μM). All chemicals are listed in Table S4.

For infections, bacterial cultures were washed twice in PBS and diluted to achieve thedesired MOI (MOI of 50 SPtA‐infected THP1 cells, MOI 15 for STm‐infected THP1 cells and MOI 100 for HeLa cell infections), which was confirmed retrospectively by colony forming unit (CFU) plating. Following infection, cells were centrifuged at 600 g for 10 min to synchronise the infection.

### 4.7 PI uptake‐dependent cell death assays

Cell media were replaced with complete media supplemented with 5 μM Propidium iodide (PI; Table S4) prior to infection. Cells were infected as described above and fluorescence was measured using a FLUOstar Omega plate reader (BMG Labtech) at 530/620 nm. Thirty minutes post infection gentamicin was added to all wells at a final concentration of 200 μg/ml and cell death was determined at 3 h post infection Experimental values were blanked with uninfected cell values and cell death was calculated as the percentage of uninfected cells treated with 0.05% Triton X‐100 (maximum lysis).

### 4.8 Gentamicin protection assays

For bacterial internalisation/invasion, THP1 cells were treated with Z‐VAD‐FMK prior to infection to prevent loss of internalised bacteria due to cell death. At 30 min post infection, cells were washed once, and media supplemented with 100 μg/ml Gentamicin was added. At 1.5 h for THP1 or at 2 h post infection for HeLa cells, cells were washed twice with PBS, lysed in 0.5% Triton X‐100 for 5 min at room temperature and serially diluted in sterile PBS. Dilutions were plated in triplicate onto LB agar plates and CFUs were enumerated.

For determining the percentage of cytosolic bacteria (escape to the cytosol), *GSDMD* ^miR^ THP1 cells were seeded and infected as above, to minimise cell death. At 30 min post infection gentamicin (100 μg/ml) was added. For the 1.5 h post infection time point, chloroquine (200 μg/ml) was additionally added to the cells for 1 h (Goddard et al., 2019), before lysing and plating CFUs as above. For the 3 h post infection time point, gentamicin was reduced to 20 μg/ml and chloroquine (200 μg/ml) was added at 2 h post infection. Cells were lysed and CFUs were determined as above.

### 4.9 Immunoblotting

THP1 cells were infected as above, except prior to infection cells were washed three times with non‐supplemented RPMI 1,640 and infections were performed in OptiMEM media supplemented with 1 mM sodium pyruvate. Supernatants were collected at 3 h post infection and precipitated at −20°C in acetone overnight, acetone was aspirated and protein supernatant and lysate samples were prepared as previously described (Eldridge et al., 2017; Goddard et al., 2019). Proteins were separated by SDS‐PAGE and transferred to 0.2 μm PVDF membrane using a TransBlot semi‐dry electrophoretic transfer machine (BioRad). Membranes were blocked in 10% milk for 1 h at room temperature and incubated at 4°C overnight with antibodies listed in Table S4. Membranes were incubated with secondary antibodies, before developing with ECL Prime using a BioRad Chemidoc Imager. Quantification of luminescent bands from western blotting was performed using the ImageLab BioRad software after subtracting background based on the UI control and by determining the ratio of the single cleaved band to the sum of the latter plus the pro‐form of each protein.

For bacterial SPI‐1 protein expression, bacteria were grown to late exponential phase and protein samples were prepared as previously described (Johnson, Byrne, et al., 2017) and transferred onto PVDF membranes as above. Antibodies used are listed in Table S4.

### 4.10 Immunofluorescence microscopy

*ASC* ^*mRFP*^ THP1 cells (Goddard et al 2019) plated onto glass coverslips were infected as described above. At 3 h post infection, cells were washed twice in PBS, fixed with 4% paraformaldehyde for 20 min at room temperature and washed thrice with PBS. Cells were quenched in 50 mM NH_4_Cl for 10 min at room temperature and permeabilised with 0.2% Triton X‐100 for 4 min. Coverslips were blocked in 2% bovine serum albumin (BSA) for 5 min before staining with the primary antibody in 2% BSA for 45 min at room temperature. Cells were washed and blocked, before adding secondary antibody, Hoechst, and Phalloidin Alexa647 (Table S4) for 30 min at room temperature. The coverslips were washed in PBS before mounting with ProLong Gold antifade reagent and visualised using a Zeiss Axio Observer Z1 microscope at × 40 magnification (Carl Zeiss).

### 4.11 Enzyme‐linked immunosorbent assay (ELISA)

THP1 cells were infected as above for 3 h and supernatants from infected cells were collected. TNF‐α or IL‐1β were measured using the ELISA kits listed in Table S4 following the manufacturer's guidelines. Sample absorbance was measured using a FLUOstar Omega plate reader (BMG Labtech) at 450 nm, and absorbance at 540 nm was subtracted for well correction.

### 4.12 Crude LPS preparation and silver staining

1.5 ml of overnight bacterial cultures were pelleted by centrifugation at 10,000 g and resuspended in 100 μl Laemmli buffer, before boiling at 100°C for 5 min. Proteinase K was added at 1 mg/ml and samples were incubated 60°C for 2 h, prior to addition of 5% β‐mercaptoethanol and further incubation for 5 min. Crude LPS samples were run on acrylamide gels, which were then fixed in 10% acetic acid/30% ethanol overnight. Gels were oxidised in oxidative solution (10% acetic acid, 30% ethanol, 1% periodic acid) for 10 min and washed three times for 15 min in water. Gels were stained for 30 min in silver stain solution (0.2 mg/ml silver nitrate) and briefly rinsed in water. Developer solution (10% acetic acid, 30% ethanol, 0.02% formaldehyde) was used to develop the stain before stopping the reaction with 1% acetic acid. Gels were handled at room temperature and incubations were performed with slow agitation.

### 4.13 RNA‐extraction and RT‐qPCR

Approximately 6 × 10^8^ bacteria were treated with RNAprotect reagent (Table S4) and digested with 15 mg/ml Lysozyme and 2 mg/ml Proteinase K for 20 min according to manufacturer's guidelines. RNA was extracted using the RNease Mini Kit following the manufacturer's instructions. Two microgram of RNA was treated with DNase as per the manufacturer's guidelines, with prolonged incubation time 1 h. Reverse transcription was performed with the MMLV transcriptase following the associated protocol. qPCR was performed using the Power Up SYBR Green master mix on the Applied Biosystems StepOnePlus system. Twenty nanogram of cDNA was used per reaction with primers listed in Table S2 at 0.2 μM final concentration. 16S was used as a reference gene (Table S2).

### 4.14 Bioinformatics and statistical analysis

Gene sequences were aligned using the ClustalW tool and visualised in JalView. Protein identity was determined using the NCBI blastp suite. No statistical methods were used to determine sample size. All experiments were repeated independently at least three times as indicated in Figure Legends. Cell death assays, gentamicin protection assays, and ELISA were performed with at least two technical repeats for each biological repeat and means from independent experiments were analysed. For immunofluorescence analysis, >100 cells were counted from randomly selected fields, % cells showing events were obtained for each biological repeat and means were compared statistically. When required, data were log‐transformed (CFU experiments) or logit‐transformed (PI death assays). Normal distribution was tested with the Shapiro–Wilk normality test. Paired two‐tailed *t*‐tests, repeated measures one‐way or two‐way ANOVA were applied to analyse data as indicated in the figure legends. Multiple comparisons were corrected by the Tukey test or the False Discovery Rate (FDR) approach of Benjamini, Krieger and Yekutieli. Statistical significance marked as: *, *p* < .05; **, *p* < .01; ***, *p* < .001; ****, *p* < .0001. All statistical analyses were performed using GraphPad Prism 8.

## CONFLICT OF INTEREST

The authors declare no competing interests.

## AUTHOR CONTRIBUTIONS

Gad Frankel, Avinash R Shenoy, and Stephen Baker Conceived the study; Elli Mylona, Julia Sanchez‐Garrido, Trang Hguyen Hoang Thu, Sabina Dongol and Abhilasha Karkey performed experimental work; Gad Frankel, Avinash R Shenoy, Stephen Baker, Elli Mylona and Julia Sanchez‐Garrido wrote the manuscript.

## Supporting information


**Appendix S1.** Supporting information.

## Data Availability

Research data not shared.
