# Supplementary material for: Very long O‐antigen chains of Salmonella Paratyphi A inhibit inflammasome activation and pyroptotic cell death
Source: Cell Microbiol. 2021 Jan 17;23(5):e13306. doi: 10.1111/cmi.13306 (PMC8609438; doi:10.1111/cmi.13306)
Supplement: Supplementary file 1 — Appendix S1. Supporting information. [file CMI-23-e13306-s001.pdf]

Figure S1. Mylona et al

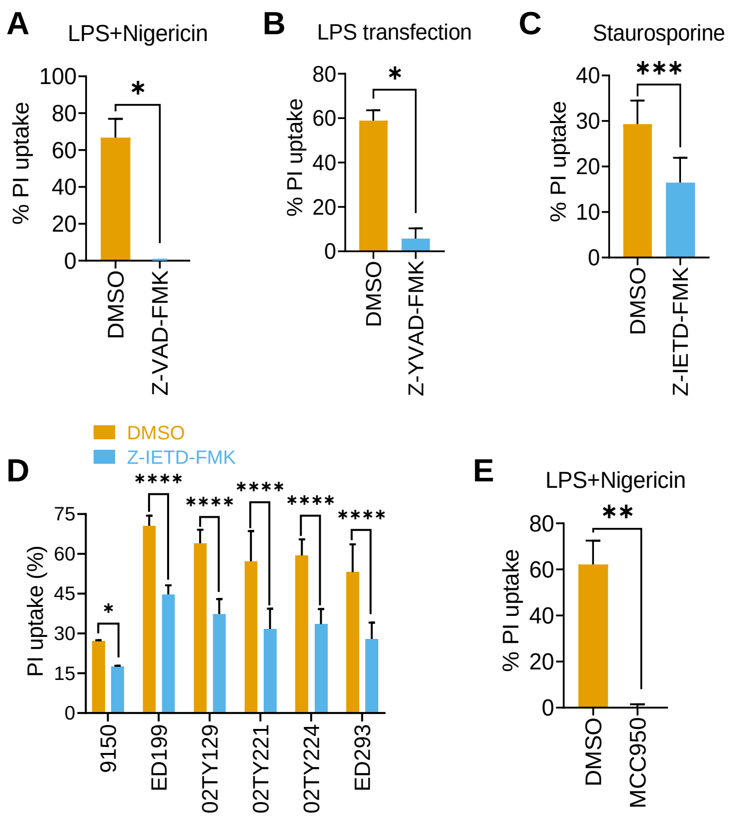

Figure S1. PI uptake into THP1 cells treated with chemical inhibitors. (A, E) THP1-cells primed with *E. coli* O111:B5 LPS were treated with either DMSO, Z-VAD-FMK **(A)** or MCC950 **(E)** and then nigericin. **(B)** THP1 cells were treated with Z-YVAD-FMK and transfected with LPS. **(C-D)** THP1 cells were treated with Z-IETD-FMK and then with Staurosporine for 5 h **(C)** or infected with the indicated SPtA strains **(D)**. Mean ± SEM from 3 **(A-D)** or 4 **(C)** independent experiments. * *P<0.05, ** P<0.01* by paired Student's *t*-test.

Figure S2. Mylona et al

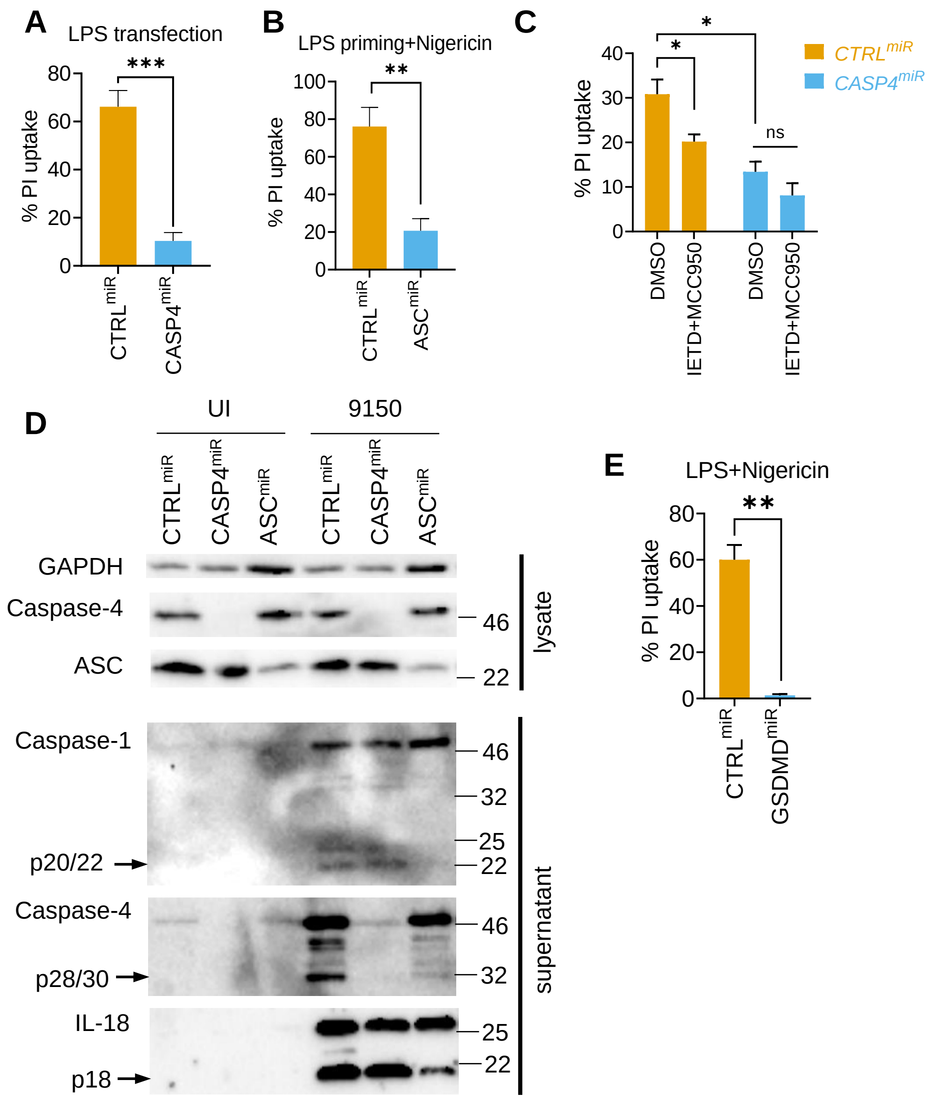

Figure S2. Functional validation of the miR THP1 cells. (A) PI uptake into CTRL^{miR} and CASP4^{miR} THP1 cells transfected with LPS. **(B)** PI uptake into CTRL^{miR} and ASC^{miR} THP1 cells primed with *E. coli* O111:B5 LPS and then treated with nigericin. **(C)** PI uptake into THP1 cells treated with DMSO or IETD plus MCC950 and infected with WT SPtA 9150. **(D)** Representative (of 2 biological repeats) immunoblots of THP1 cell lines left uninfected (UI) or infected with WT SPtA 9150 for cleaved products of caspase-1, caspase-4 and IL-18 (arrows). **(E)** PI uptake into CTRL^{miR} and GSDMD^{miR} THP1 cells primed with *E. coli* O111:B5 LPS followed by nigericin treatment. Mean ± SEM means from 6 **(A),** or 4 **(B, C, E)** independent experiments are shown. ** *P<0.01,* *** *P<0.001,* for comparisons by paired Student's *t*-test.

Figure S3. Mylona et al

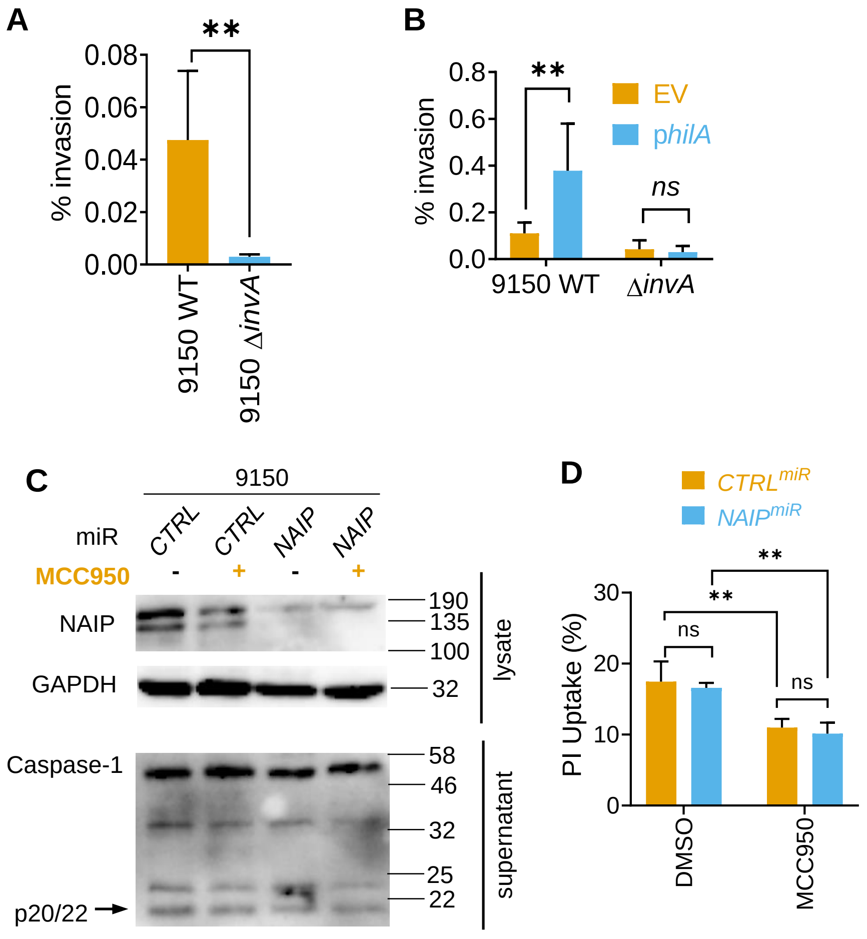

Figure S3. SPI-1 T3SS is required for HeLa cell invasion by SPtA. (A-B) Invasion of HeLa cells by the indicated SPtA 9150 strains. **(C)** Representative (of 3 independent repeats) immunoblots of THP1 cell lines left uninfected (UI) or infected with WT SPtA 9150 in the absence or presence of MCC950 for cleaved products of caspase-1 (arrow). **(D)** PI uptake assays in the indicated THP1 cells treated with DMSO or MCC950 and infected with WT SPtA 9150 for 3 h. Mean ± SEM from 4 **(A-B)** or 3 **(D)** independent experiments. ** *P<0.01* by **(A)** paired Student's *t*-test or **(B, D)** matched two-way ANOVA after correction for multiple comparisons; ns, not significant.

Figure S4. Mylona et al

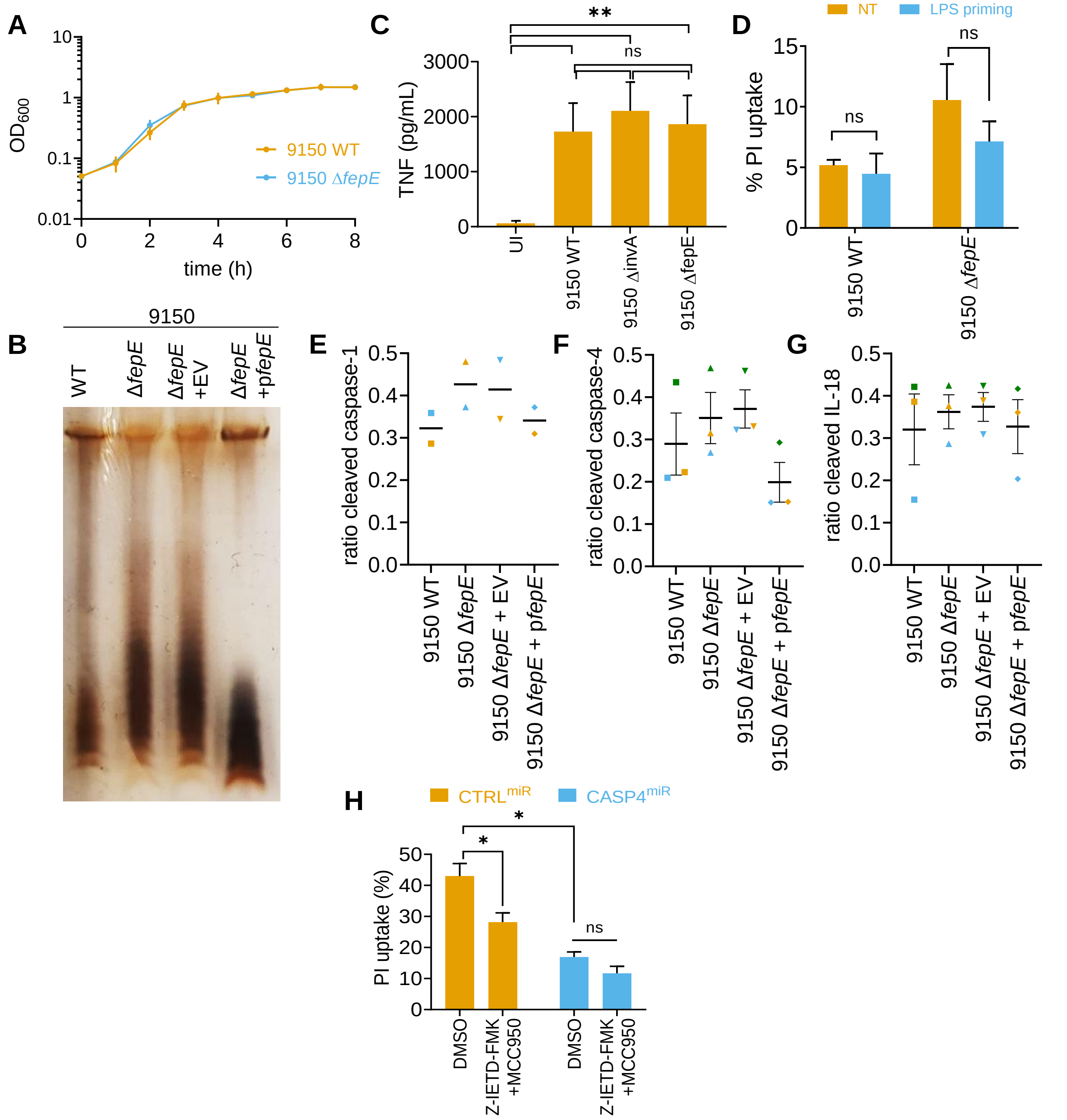

Figure S4. The effect of loss of very long LPS O-antigen chains on THP1 priming. (A) Growth curves of WT SPtA 9150 and Δ*fepE* as measured by OD_{600}. **(B)** Representative (of 2 biological repeats) image of silver-stained acrylamide gel showing electrophoretic mobility of LPS extracted from the indicated strains of SPtA. **(C)** ELISA quantification of TNF in culture supernatants of THP1 cells infected with the indicated SPtA strains. **(D)** PI uptake into unprimed THP1 cells (NT) or primed with *E. coli* O111:B5 LPS before infection with WT SPtA 9150 or Δ*fepE*. **(E-G)** Quantification of luminescent bands of western blots of caspase-1 (E), caspase-4 (F), and IL-18 (G) showing the ratio of the cleaved protein to the sum of cleaved plus pro-form of each protein in the supernatant of THP-1 cells infected with the indicated SPtA strains. **(H)** PI uptake into $CTRL^{miR}$ or $CASP4^{miR}$ THP1 cells infected with SPtA in the presence of DMSO alone or Z-IETD-FMK plus MCC950. Mean ± SEM from 2 **(A)**, 3 **(B, E-G)** or 4 **(D, H)** independent experiments. * *P<0.05*, ** *P<0.01* for indicated comparisons by matched one-way ANOVA **(C, E-G)** or two-way ANOVA **(D, H)** after correction for multiple comparisons; ns, not significant.

Figure S5. Mylona et al

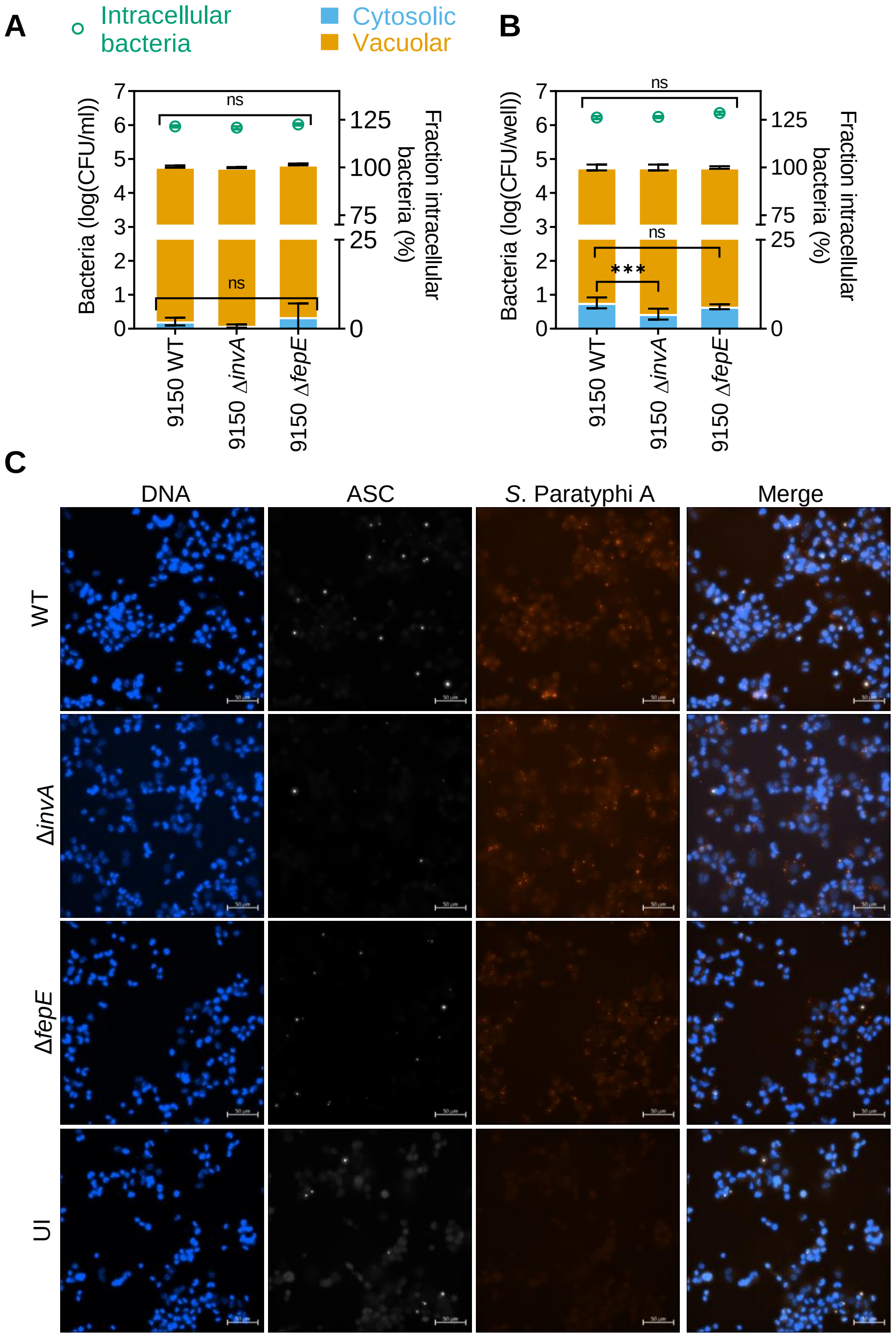

Figure S5. The role of very long O-antigen chains in vacuolar escape and ASC speck formation. (A-B) Bacterial internalisation (left-axis) or relative cytosolic escape measured by chloroquine-resistance assay (right-axis) of the indicated SPtA strains into GSDMD^{miR} THP-1 cells at 1.5 h **(A)** or 3 h **(B)**. **(C)** Representative (of 4 biological repeats) images from THP1-ASC^{mRFP} cells left uninfected (UI) or infected with the indicated SPtA strains. Scale bar 50 µm. Means ± SEM from 5 **(A)** or 3 **(B)** independent repeats are depicted. *** *P<0.001* by matched one-way ANOVA after correction for multiple comparisons; ns, not significant for comparison of WT SPtA with other strains.

Figure S6. Mylona et al

```
SPtA    1 MPSLNVKQEKNQSFAGYSLPPANSHEIDLFSLIEVLWQAKRRILATVFAFACV  53
STm     1 MPSLNVKQEKNQSFAGYSLPPANSHEIDLFSLIEVLWQAKRRILATVFAFACV  53

SPtA   54 GLLLSFLLPQKWTSQAIVTPAESVQWQGLERTLTALRVLDMEVSVDRGSVFNL 106
STm    54 GLLLSFLLPQKWTSQAIVTPAESVQWQGLERTLTALRVLDMEVSVDRGSVFNL 106

SPtA  107 FIKKFSSPSLLEEYLRSSPYVMDQLKGAQIDEQDLHRAIVLLSEKMKAVDSNV 159
STm   107 FIKKFSSPSLLEEYLRSSPYVMDQLKGAQIDEQDLHRAIVLLSEKMKAVDSNV 159

SPtA  160 GKKNETSLFTSWTLSFTAPTREEAQKVLAGYIQYISDIVVKETLENIRNQLEI 212
STm   160 GKKNETSLFTSWTLSFTAPTREEAQKVLAGYIQYISDIVVKETLENIRNQLEI 212

SPtA  213 KTRYEQEKLAMDRVRLKNQLDANIQRLHYSLEIANAAGIKRPVYSNGQAVKDD 265
STm   213 KTRYEQEKLAMDRVRLKNQLDANIQRLHYSLEIANAAGIKRPVYSNGQAVKDD 265

SPtA  266 PDFSISLGADGISRKLEIEKGVTDVAEIDGDLRNRQYHVEQLAAMNVSDVKFT 318
STm   266 PDFSISLGADGISRKLEIEKGVTDVAEIDGDLRNRQYHVEQLAAMNVSDVKFT 318

SPtA  319 PFKYQLSPSLPVKKDGPGKAIIIILAALIGGMMACGGVLLRHAMVSRKMENAL 371
STm   319 PFKYQLSPSLPVKKDGPGKAIIIILAALIGGMMACGGVLLHHAMVSRKMENAL 371

SPtA  372 AIDERLV 378
STm   372 AIDERLV 378
```

Figure S6. Protein sequence alignment of SPtA and STm *fepE*. Amino acid sequence of SPtA 9150 *fepE* and STm *fepE*; amino acid differences are depicted in red.

Table S1. Strains and plasmids used in this study

Strain or plasmid name	Source	Identifier
**Strains**		
Salmonella enterica serovar Paratyphi A ATCC 9150 (SPtA 9150) WT	(Gal-Mor et al., 2012)	N/A
SPtA ED199	(Dolecek et al., 2008)	N/A
SPtA 02TY129	(Arjyal et al., 2016)	N/A
SPtA 02TY221	(Arjyal et al., 2016)	N/A
SPtA 02TY224	(Arjyal et al., 2016)	N/A
SPtA ED293	(Dolecek et al., 2008)	N/A
SPtA 9150 Δ*fepE*::Kan^r	This study	ICC 1913
SPtA 9150 Δ*invA*::Kan^r	This study	ICC 1874
Salmonella enterica serovar Typhimurium 14028 (STm) WT	(Johnson et al., 2018)	ICC 797
STm Δ*fepE*::Kan^r	This study	
**Plasmids**		
pWS29K-Spec EV	(Johnson et al., 2017)	pICC2489
pWSK29-Spec-*hilA*	This study	pICC2767
pWSK29-Spec-*fepE*	This study	pICC2768

Table S2. Primers used in this study

Primer name	Source	Identifier
$^{5'}$TCATTGGATAAAGTTTTCAGGTCATACGGCATGCCATCTCTTAATGTAAAACTGTGTAGGCTGGAGCTGCTTCG$^{3'}$	This study (for generating Δ*fepE*)	N/A
$^{5'}$CATATGAATATCCTCCTTAGGGATATCGCTATCCGGCTTTTCGGGTAAATCAGACTAACCGTTCATCTA$^{3'}$	This study (for generating Δ*fepE*)	N/A
$^{5'}$AGGCAAGTTTAATTCCGAATATTTAGC$^{3'}$	This study (for sequencing *fepE* deletion)	N/A
$^{5'}$TATGGGCTGCGCTGTATGATTATC$^{3'}$	This study (for sequencing *fepE* deletion)	N/A
$^{5'}$ACTTAACAGTGCTCGTTTACGACCTGAATTACTGATTCTGGTACTAATGGGTGTAGGCTGGAGCTGCTTC$^{3'}$	This study (for generating Δ*invA*)	(Johnson et al., 2017)
$^{5'}$GCTATCTGCTATCTCACCGAAAGATAAAACCTCCAGATCCGGAAAACGACCCATATGAATATCCTCCTTAG$^{3'}$	This study (for generating Δ*invA*)	(Johnson et al., 2017)
$^{5'}$GTTACGTAGTATGACCGATATTGC$^{3'}$	This study (for sequencing *invA* deletion)	(Johnson et al., 2017)
$^{5'}$CCTCCAATTAATGAAGGATCGC$^{3'}$	This study (for sequencing *invA* deletion)	(Johnson et al., 2017)
$^{5'}$GCTCCACCGCGGTGGCGGCCATGCCACATTTTAATCCTG$^{3'}$	This study (for generating pWSK29-Spec-*hilA*)	N/A
$^{5'}$GGATCCACTAGTTCTAGAGCTTACCGTAATTTAATCAAGCG$^{3'}$	This study (for generating pWSK29-Spec-*hilA*)	N/A
$^{5'}$GCTCTAGAACTAGTGGATC$^{3'}$	This study (for generating pWSK29-Spec-*hilA*)	N/A
$^{5'}$GGCCGCCACCGCGGTGGA$^{3'}$	This study (for generating pWSK29-Spec-*hilA*)	N/A
$^{5'}$CTCCACCGCGATGCCATCTCTTAATGTAAAAC$^{3'}$	This study (for generating pWSK29-Spec-*fepE*)	N/A
$^{5'}$TACCGGGCCCTCAGACTAACCGTTCATC$^{3'}$	This study (for generating pWSK29-Spec-*fepE*)	N/A
$^{5'}$GTTAGTCTGAGGGCCCGGTACCCAATTC$^{3'}$	This study (for generating pWSK29-Spec-*fepE*)	N/A
$^{5'}$GAGATGGCATCGCGGTGGAGCTCCAGCT$^{3'}$	This study (for generating pWSK29-Spec-*fepE*)	N/A
$^{5'}$GGGTTGCTTCTGTCCTTTCTG $^{3'}$	This study (RT-qPCR for *fepE*)	N/A

$^{5'}$TTACCTCCATATCCAACACGC$^{3'}$	This study (RT-qPCR for *fepE*)	N/A
$^{5'}$TGTAGCGGTGAAATGCGTAG$^{3'}$	This study (RT-qPCR for *16S*)	N/A
$^{5'}$CAAGGGCACAACCTCCAAG$^{3'}$	This study (RT-qPCR for *16S*)	N/A

Table S3. Cell lines (A) and oligopeptides (B) used in this study

**(A) Cell line name**	**Source**	**Identifier**
THP-1	John MacMicking laboratory (Shenoy et al., 2012)	N/A
HeLa	ATCC	ATCC CCL-2
**(B) Oligopeptide name**	**Source**	**Identifier**
pMX-CMV-YFP-LacZ^{miR} $^{5'}$-TCACGACGTTGT AATACGACGT-$^{3'}$	(Eldridge et al., 2017)	N/A
pMX-mAsc-mRFP	(Goddard et al., 2019)	N/A
pMX-CMV-YFP-GSDMD^{miR} $^{5'}$-TACACATTCATTGAGGTGCTGG-$^{3'}$	(Eldridge et al., 2017)	N/A
pMX-CMV-YFP-CASP4^{miR} $^{5'}$-ATATCTTGTCATGGACAGTCGT-$^{3'}$	(Goddard et al., 2019; Pallett et al., 2016)	N/A
pMX-CMV-YFP-ASC^{miR} $^{5'-}$CAGCTCTTCAGTTTCACACCAG-$^{3'}$	(Goddard et al., 2019)	N/A
pMX-CMV-YFP-NAIP^{miR} $^{5'}$-ATTCACAAAGTTCACCACGGCT-$^{3'}$	This study	N/A

Table S4. Chemicals, reagents and antibodies used in this study

Chemical/Reagent name	Source	Identifier
Mouse anti-human Caspase-4 (4B9) antibody	Santa Cruz Biotechnology	Cat# sc-56056, RRID:AB_781828
anti-Caspase-1 (p20) (human) mAb (Bally-1) antibody	AdipoGen	Cat# AG-20B-0048, RRID:AB_2490257
Human IL-18 Polyclonal Antibody	MBL International	Cat# PM014, RRID:AB_592017
anti-Asc pAb (AL177) antibody	AdipoGen	Cat# AG-25B-0006, RRID:AB_2490440
Mouse Anti-Human GSDMDC1 Monoclonal Antibody, Unconjugated, Clone 64-Y	Santa Cruz Biotechnology	Cat# sc-81868, RRID:AB_2263768
Mouse anti-human NAIP	R&D Systems	Cat# MAB829
Rabbit anti-GAPDH	Abcam	Cat# ab9485
DnaK (E. coli), mAb (8E2/2) antibody	Enzo Life Sciences	Cat# ADI-SPA-880, RRID:AB_10619012
Rabbit anti-SipA (Salmonella)	V Koronakis Lab (Johnson et al., 2017)	N/A
Rabbit anti-SipC (Salmonella)	V Koronakis Lab (Johnson et al., 2017)	N/A
Rabbit anti-SipD (Salmonella)	V Koronakis Lab (Johnson et al., 2017)	N/A
Goat anti-CSA-1	Insight Biotechnology	Cat# 01-91-99
Peroxidase AffiniPure Goat Anti-Mouse IgG, Fcγ fragment specific	Jackson Immunoresearch	Cat# 115-035-008 RRID: AB_2313585
Peroxidase AffiniPure Goat Anti-Rabbit IgG, Fc fragment specific	Jackson Immunoresearch	Cat# 111-035-008 RRID: AB_2337937
Donkey anti-goat immunoglobulin-Cy2	Jackson ImmunoResearch	Cat# 705-225-147 RRID: AB_2307341
Hoechst 33258	Sigma-Aldrich	Cat# 94403
Phalloidin Alexa647	Startech	Cat# 23127-AAT
LPS-EB (LPS from E. coli O111:B4)	Invivogen	Cat# tlrl-3pelps
Chloroquine	Sigma-Aldrich	Cat# C6628
Nigericin	Sigma-Aldrich	Cat# N7143
Kanamycin	Sigma-Aldrich	Cat# 60615
Spectinomycin dihydrochloride pentahydrate	Sigma-Aldrich	Cat# S9007
Puromycin dihydrochloride from Streptomyces alboniger	Sigma-Aldrich	Cat# P8833

Gentamicin	Sigma-Aldrich	Cat # G1272
MCC950	Tocris Bioscience	Cat # 5479
Lipofectamine 2000 Transfection Reagent	Life Technologies	Cat# 11668027
Pierce Protease Inhibitor Mini Tablets, EDTA-free	Thermo Fisher Scientific	Cat# A32955
ECL Prime Western Blotting Detection Reagent	GE-Healthcare	Cat# RPN2236
Propidium iodide (PI)	Sigma-Aldrich	Cat # P4864
ProLong Gold Antifade Mountant	Thermo Fisher Scientific	Cat# P36930
Phenylmethanesulfonyl fluoride	Sigma-Aldrich	Cat# P7626
Phorbol myristate acetate (PMA)	Sigma-Aldrich	Cat# P8139
HEPES solution	Sigma-Aldrich	Cat# H0887
Trypsin	Sigma-Aldrich	Cat# T4674
EDTA 0.2% cell culture	Sigma-Aldrich	Cat# E8008
Dulbecco’s minimal Eagle media High Glucose (4500mg/L)	Sigma-Aldrich	Cat# D5796
RPMI 1640	Sigma-Aldrich	Cat# R8758
RPMI 1640 – Phenol Red Free	Gibco	Cat# 11835030
Fetal Bovine Serum	Gibco	Cat# 10270-106
Sodium pyruvate	Sigma-Aldrich	Cat# S8636
Penicillin-Streptomycin	Sigma-Aldrich	Cat# 31985062
GlutaMAX supplement	ThermiFisher Scientific	Cat# 35050061
Gibson assembly master mix	NEB	Cat# E2611
Z-VAD-FMK	R&D Systems	Cat# FMK001
Z-YVAD-FMK	R&D Systems	Cat# FMK001
Z-IETD-FMK	R&D Systems	Cat# FMK007
Staurosporine	Sigma-Aldrich	Cat# S5921
Paraformaldehyde (PFA)	ThermoFisher Scientific	Cat# 28908
TNF alpha Human Uncoated ELISA Kit	ThermoFisher Scientific	Cat# 88-7346-22
Power Up SYBR Green	Applied Biosystems	Cat# 15350929
MycoAlert mycoplasma detection kit	Lonza	Cat# LT07-518
IL-1beta Human Uncoated ELISA Kit	ThermoFisher Scientific	Cat# 88-7261-22
RNAprotect	Qiagen	Cat# 76526
RNeasy Mini Kit	Qiagen	Cat# 74106
MMLV reverse trancriptase	Promega	Cat# M1706

DNase	Promega	Cat# M6101
Lysozyme	Sigma	Cat# L6876

Arjyal, A., Basnyat, B., Nhan, H. T., Koirala, S., Giri, A., Joshi, N., Shakya, M., Pathak, K. R., Mahat, S. P., Prajapati, S. P., Adhikari, N., Thapa, R., Merson, L., Gajurel, D., Lamsal, K., Lamsal, D., Yadav, B. K., Shah, G., Shrestha, P., … Dolecek, C. (2016). Gatifloxacin versus ceftriaxone for uncomplicated enteric fever in Nepal: An open-label, two-centre, randomised controlled trial. *The Lancet Infectious Diseases*, *16*(5), 535–545. https://doi.org/10.1016/S1473-3099(15)00530-7

Dolecek, C., La, T. T. P., Rang, N. N., Phuong, L. T., Vinh, H., Tuan, P. Q., Du, D. C., Bay, N. T. B., Long, D. T., Ha, L. B., Binh, N. T., Hong, N. T. A., Dung, P. N., Lanh, M. N., Van Be Bay, P., Ho, V. A., Van Minh Hoang, N., Nga, T. T. T., Chau, T. T., … Farrar, J. (2008). A multi-center randomised controlled trial of gatifloxacin versus azithromycin for the treatment of uncomplicated typhoid fever in children and adults in Vietnam. *PLoS ONE*, *3*(5). https://doi.org/10.1371/journal.pone.0002188

Eldridge, M. J. G., Sanchez-Garrido, J., Hoben, G. F., Goddard, P. J., & Shenoy, A. R. (2017). The Atypical Ubiquitin E2 Conjugase UBE2L3 Is an Indirect Caspase-1 Target and Controls IL-1β Secretion by Inflammasomes. *Cell Reports*, *18*(5), 1285–1297. https://doi.org/10.1016/j.celrep.2017.01.015

Gal-Mor, O., Suez, J., Elhadad, D., Porwollik, S., Leshem, E., Valinsky, L., McClelland, M., Schwartz, E., & Rahav, G. (2012). Molecular and Cellular Characterization of a Salmonella enterica Serovar Paratyphi A Outbreak Strain and the Human Immune Response to Infection. *Clinical and Vaccine Immunology*, *19*(2), 146–156. https://doi.org/10.1128/cvi.05468-11

Goddard, P. J., Sanchez-Garrido, J., Slater, S. L., Kalyan, M., Ruano-Gallego, D., Marchès, O., Fernández, L. Á., Frankel, G., & Shenoy, A. R. (2019). Enteropathogenic Escherichia coli Stimulates Effector-Driven Rapid Caspase-4 Activation in Human Macrophages. *Cell Reports*, *27*(4), 1008-1017.e6. https://doi.org/10.1016/j.celrep.2019.03.100

Johnson, R., Byrne, A., Berger, C. N., Klemm, E., Crepin, V. F., Dougan, G., & Frankel, G. (2017). The type III secretion system effector SptP of Salmonella enterica serovar Typhi. *Journal of Bacteriology*, *199*(4), JB.00647-16. https://doi.org/10.1128/JB.00647-16

Johnson, R., Ravenhall, M., Pickard, D., Dougan, G., Byrne, A., & Frankel, G. (2018). Comparison of Salmonella enterica serovars Typhi and Typhimurium reveals typhoidal serovar-specific responses to bile. *Infection and Immunity*, *86*(3), IAI.00490-17. https://doi.org/10.1128/IAI.00490-17

Pallett, M., Crepin, V., Serafini, N., Habibzay, M., Kotik, O., Sanchez-Garrido, J., Santo, D., Shenoy, A., Berger, C., & Frankel, G. (2016). Bacterial virulence factor inhibits caspase-4/11 activation in intestinal epithelial cells. *Nature Publishing Group*, *10*. https://doi.org/10.1038/mi.2016.77

Shenoy, A. R., Wellington, D. A., Kumar, P., Kassa, H., Booth, C. J., Cresswell, P., & MacMicking, J. D. (2012). GBP5 Promotes NLRP3 Inflammasome Assembly and Immunity in Mammals. *Science*, *336*(6080), 481–485. https://doi.org/10.1126/science.1217032
